# Real-Time Genomic Surveillance for SARS-CoV-2 Variants of Concern, Uruguay

**DOI:** 10.3201/eid2711.211198

**Published:** 2021-11

**Authors:** Natalia Rego, Alicia Costábile, Mercedes Paz, Cecilia Salazar, Paula Perbolianachis, Lucía Spangenberg, Ignacio Ferrés, Rodrigo Arce, Alvaro Fajardo, Mailen Arleo, Tania Possi, Natalia Reyes, Ma Noel Bentancor, Andrés Lizasoain, María José Benítez, Viviana Bortagaray, Ana Moller, Gonzalo Bello, Ighor Arantes, Mariana Brandes, Pablo Smircich, Odhille Chappos, Melissa Duquía, Belén González, Luciana Griffero, Mauricio Méndez, Ma Pía Techera, Juan Zanetti, Bernardina Rivera, Matías Maidana, Martina Alonso, Cecilia Alonso, Julio Medina, Henry Albornoz, Rodney Colina, Veronica Noya, Gregorio Iraola, Tamara Fernández-Calero, Gonzalo Moratorio, Pilar Moreno

**Affiliations:** Institut Pasteur, Montevideo, Uruguay (N. Rego, A. Costábile, M. Paz, C. Salazar, P. Perbolianachis, L. Spangenberg, I. Ferres, R. Arce, A. Fajardo, M. Brandes, B. Rivera, M. Maidana, M. Alonso, G. Iraola, T. Fernandez-Calero, G. Moratorio, P. Moreno);; Universidad de la República, Montevideo, Uruguay (A. Costabile, P. Perbolianachis, R. Arce, A. Fajardo, A. Lizasoain, M.J. Benítez, V. Bortagaray, A. Moller, O. Chappos, M. Duquía, B. González, L. Griffero, M. Méndez, M.P. Techera, J. Zanetti, C. Alonso, R. Colina, G. Moratorio, P. Moreno);; Sanatorio Americano, Montevideo (M. Arleo, T. Possi, N. Reyes, M.N. Bentancor, V. Noya);; Instituto Oswaldo Cruz–Fiocruz, Rio de Janeiro, Brazil (G. Bello, I. Arantes);; Instituto de Investigaciones Biológicas Clemente Estable, Montevideo (P. Smirnich);; Ministerio de Salud Pública, Montevideo (J. Medina, H. Albornoz)

**Keywords:** genomic surveillance, epidemiology, PCR, variants of concern respiratory infections, severe acute respiratory syndrome coronavirus 2, SARS-CoV-2, SARS, COVID-19, coronavirus disease, zoonoses, viruses, coronavirus, Uruguay

## Abstract

We developed a genomic surveillance program for real-time monitoring of severe acute respiratory syndrome coronavirus 2 (SARS-CoV-2) variants of concern (VOCs) in Uruguay. We report on a PCR method for SARS-CoV-2 VOCs, the surveillance workflow, and multiple independent introductions and community transmission of the SARS-CoV-2 P.1 VOC in Uruguay.

By late 2020, because of natural viral evolution, severe acute respiratory syndrome coronavirus 2 (SARS-CoV-2) genetic variants emerged, some of which show increased transmissibility and cause more severe coronavirus disease (COVID-19) (*1*). In addition, these variants show reduced neutralization by antibodies generated during previous infection or vaccination, which can reduce effectiveness of treatments, vaccines, or diagnostic tests (*1*). By July 2021, a total of 4 variants of concern (VOCs) had been identified: B.1.1.7 (Alpha), B.1.351 (Beta), P.1 (Gamma), and B.1.617 (Delta) (*2*). Nonetheless, a robust surveillance workflow for early VOC identification is key to accelerating the pandemic response.

Brazil demonstrated a sharp increase in SARS-CoV-2 cases, hospitalizations, and deaths after the emergence of the P.1 VOC in Amazonas State in November 2020 (*3*; F. Naveca et al., unpub. data, https://doi.org/10.21203/rs.3.rs-275494/v1). P.1 displays higher transmissibility than previous local SARS-CoV-2 lineages and rapidly became the predominant strain in most states of Brazil during February–March 2021 (*3*,*4*; F. Naveca et al., unpub. data, https://doi.org/10.21203/rs.3.rs-275494/v1). P.1 also has spread worldwide; by July 2021, P.1 had been detected in >41 countries (*5*), where it might replicate the epidemic trajectory observed in Brazil. Uruguay, which shares 600 miles of dry border with Brazil, has experienced an exponential increase in COVID-19 cases since February 2021; by June 2021, Uruguay was among countries with the highest number of daily cases and deaths per million persons (*6*). Despite closing the Brazil–Uruguay border to tourism on March 13, 2020, evidence suggests a high viral flux between the countries (*7*,*8*). Therefore, P.1 could be introduced into Uruguay and the country needs an organized strategy to monitor VOC emergence. 

In response to concerns over VOCs, the Ministry of Public Health, the Pasteur Institut of Montevideo (Uruguay), University of the Republic, and Zurgen-Sanatorio Americano formed a multidisciplinary workgroup to develop a genomic surveillance program for real-time monitoring for VOC emergence in Uruguay. The workgroup aimed to provide expertise and resources for large-scale sequencing, genomic analysis, and an affordable and decentralized inhouse PCR to detect known VOCs, including B.1.1.7, B.1.351, and P.1. Within a few weeks, the working group developed a PCR VOC detection method and a national sample processing workflow (Appendix 1 Figure 1). In addition, we identified multiple independent introductions of P.1 and community transmission in Uruguay.

The workgroup processes >3,000 nasopharyngeal samples daily, and around 100–300 SARS-CoV-2–positive samples are sent weekly for PCR VOC analysis and sequencing. During January 11–March 26, 2021, the working group collected and processed a total of 251 SARS-CoV-2–positive RNA samples from 15/19 departments in Uruguay (Appendix 1 Table 1). Cycle thresholds for initial diagnostic PCR were 9–34.7. Among patients with positive samples, 95 were male, 95 were female, and 61 were of unknown sex; ages ranged from 1–85 years. Results from PCR VOC assay showed that 67/251 (27%) samples corresponded to putative P.1/B.1.351 (Appendix 1 Figure 2). 

To validate PCR VOC classification, we sequenced all VOC–positive samples, plus 31 additional samples, by applying the ARTIC Network protocol (J.R. Tyson et al., unpub. data, https://doi.org/10.1101/2020.09.04.283077) for the MinION sequencing platform (Oxford Nanopore Technologies, https://nanoporetech.com) (Appendix 1). For the final 74 high-quality consensus sequences that were assigned to SARS-CoV-2 lineages following Pango nomenclature (*9*), we achieved a 100% agreement between PCR VOC and genome sequencing results. Predictably, given Uruguay’s proximity to Brazil, samples classified as P.1/B.1.351 by PCR VOC were assigned to lineage P.1 after genome sequencing. The PCR we developed is a feasible, precise, and scalable method for real-time surveillance of known VOCs and verified circulation of P.1 lineage in 15/19 departments in Uruguay (Appendix 1 Figures 2, 3). 

To estimate geographic sources and the number of independent P.1 introductions into Uruguay, we combined P.1 sequences from Uruguay and 691 P.1 sequences from South America available from EpiCoV in GISAID (https://www.epicov.org/epi3; Appendix 1 Tables 2, 3; Appendix 2). The maximum-likelihood phylogeographic analysis identified >12 independent P.1 introductions into Uruguay from Brazil and >6 local transmission clusters of 3–24 sequences (Figure; Appendix 1 Figure 4). We used Bayesian analysis to estimate the median time of most recent common ancestor of P.1 clades in Uruguay to be mid-February to early March 2021 (Appendix 1 Table 4, Figure 5), which coincides with increasing mobility and the beginning of the exponential surge in COVID-19 cases in the country (Appendix 1 Figure 6).

**Figure:**
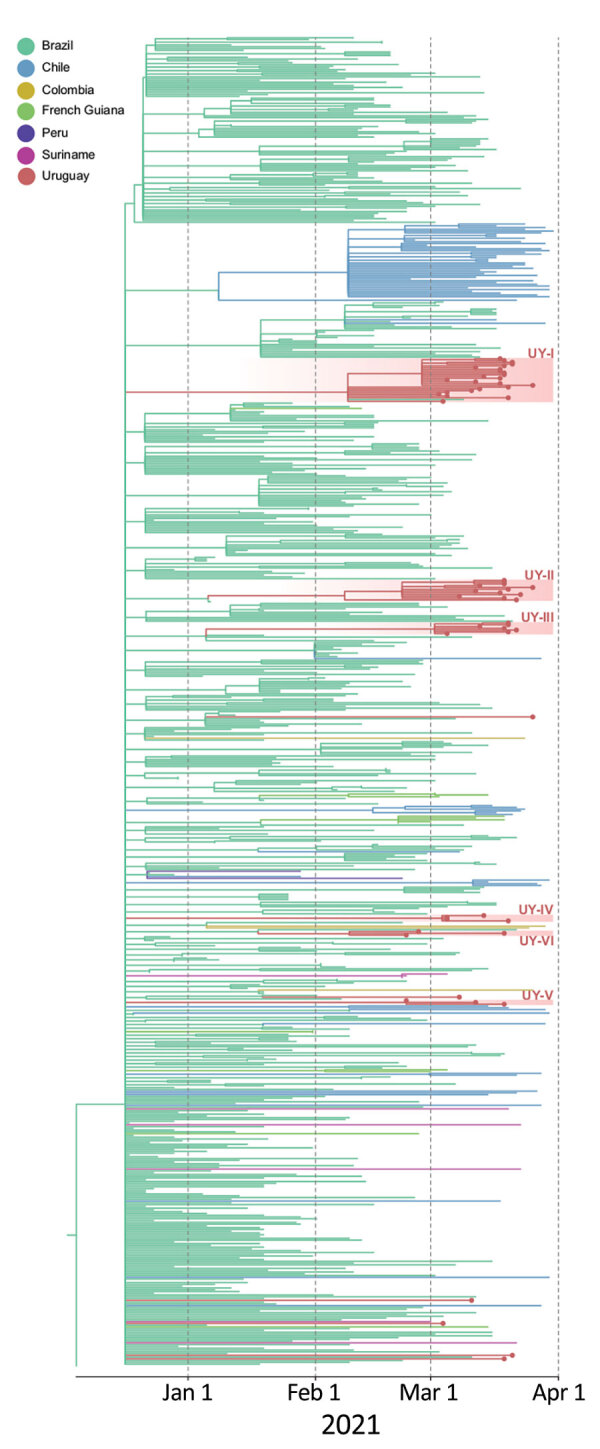
Time-scaled maximum likelihood Bayesian phylogeographic maximum clade credibility tree of 59 severe acute respiratory syndrome coronavirus 2 lineage P.1 whole-genome sequences from Uruguay and 691 reference sequences from South America. The tree was rooted with the EPI_ISL_833137 sequence from GISAID (https://www.gisaid.org), collected December 4, 2020. Branches are colored according to the most probable location state of their descendant nodes as indicated at the legend. Sequences from Uruguay are shown with dots at the end of the branch. Red shading indicates clades from Uruguay and their distribution along the P.1 tree demonstrates >12 independent introductions and locally transmitted clusters of 3–24 sequences. The tree suggests Brazil has been the source of P.1 dissemination to Uruguay and other countries in South America.

The rapid emergence of the SARS-CoV-2 lineage P.1 in South America justifies the need for increased screening for this highly transmissible virus. We elaborated a comprehensive genomic surveillance program and provide a clear example of how multidisciplinary teams are key mechanisms for helping public health authorities manage the COVID-19 crisis. Our findings revealed that the P.1 VOC was introduced into Uruguay multiple times over a period of increasing mobility in binational cities along the Brazil–Uruguay border and in Uruguay between mid-February and early March 2021. The introduction of the highly transmissible P.1 VOC coupled with the increasing human mobility probably contributed to the rapid local spread of this variant and the worsening COVID-19 epidemic in Uruguay during January–July 2021.

Appendix 1 Additional information on real-time genomic surveillance for SARS-CoV-2 variants of concern, Uruguay.

Appendix 2 Acknowledgments for SARS-CoV-2 sequences downloaded from EpiCoV database in the GISAID initiative for a study of variants of concern, Uruguay.
