# Supplementary material for: Real-Time Genomic Surveillance for SARS-CoV-2 Variants of Concern, Uruguay
Source: Emerg Infect Dis. 2021 Nov;27(11):2957–60. doi: 10.3201/eid2711.211198 (PMC8544970; doi:10.3201/eid2711.211198)
Supplement: Appendix 1 — Additional information on real-time genomic surveillance for SARS-CoV-2 variants of concern, Uruguay. [file 21-1198-Techapp-s1.pdf]

Article DOI: https://doi.org/10.3201/eid2711.211198

Real-Time Genomic Surveillance for SARS-CoV-2 Variants of Concern, Uruguay

Appendix 1

Supplementary Methods

Residual deidentified RNA samples from severe acute respiratory syndrome coronavirus 2 (SARS-CoV-2)–positive patients were remitted to the Institut Pasteur Montevideo, which is an approved center providing diagnostic testing for coronavirus disease (COVID-19) validated by the Ministry of Health of Uruguay. All samples were deidentified before receipt by the study investigators.

Drop Out PCR for SARS-CoV-2 Variants of Concern

We designed primers and drop out probes to target the deletion sites in both spike (S) and open reading frame 1ab (ORF1ab) viral genes for this assay. We used the nucleotide sequence from a Wuhan SARS-CoV-2 strain (GenBank acc. NC_045512) to devise primers and probes targeting ORF1ab deletion Δ3675–3677, which causes a spike gene target failure (Appendix 1 Table 5). We used the OligoAnalyzer Tool (Integrated DNA Technologies, https://www.idtdna.com; *1*) to assess oligonucleotide complementarity and melting temperature. We used primers and probes from C.B.F. Vogels et al. (*2*) that target S deletion in amino acid 69 histidine and 70 valine positions (Δ69–70 HV) (Appendix 1 Table 5). B.1.1.7 has both the Δ69–70 HV and Δ3675–3677 deletions, but P.1 and B.1.351 have only the Δ3675–3677 deletion in ORF1ab. Hence, we expected no amplification with both probes with B.1.1.7 lineage viruses, only amplification of the S gene with P.1 and B.1.351 lineages and amplification of both genomic regions with other lineages. We used BLAST (NCBI, https://blast.ncbi.nlm.nih.gov) to analyze the primer and probe sets to rule out similarities with sequences other than SARS-CoV-2. We used the standard hydrolysis probe system from TaqMan technology as the basis of the detection strategy. We labeled the S gene–specific probes with HEX fluorophore (Integrated DNA Technologies) and custom labeled the Orf1a probe with CY5 fluorophore (Integrated DNA

Technologies). Primers and probes were purified by high-pressure liquid chromatography. We prepared the reaction mix by using 5 µL of RNA samples, 5 µL of 4× TaqMan Fast Virus 1-Step Master Mix (Thermo Fisher Scientific, https://www.thermofisher.com), with 0.3 µmol of each S primer, 0.1 µmol of S probe, 0.3 µmol of each Orf1a primer, 0.1 µmol of Orf1a probe, and molecular biology grade water to a final volume of 20 µL. We performed thermal cycling on a QuantStudio 7 Pro real-time PCR system (Applied Biosystems, https://www.thermofisher.com) with the following cycle parameters: 50°C for 5 min for reverse transcription, inactivation of reverse transcription at 95°C for 20 s, and then 40 cycles at 95°C for 15 s and 60°C for 30 s.

Genome Sequencing

We prepared sequencing libraries according to the Eco PCR tiling of COVID-19 virus protocol (Oxford Nanopore Technologies), according to the method described by J. Quick (*3*; J.R. Tyson et al., unpub. data, https://doi.org/10.1101/2020.09.04.283077) with some modifications. For RNA samples previously screened by a PCR assay, we used SuperScript II Reverse Transcriptase Kit (Thermo Fisher Scientific Inc.) or the LunaScript RT SuperMix Kit (New England Biolabs, https://www.neb.com) to perform reverse transcription. We included a negative control at this point and carried it throughout the protocol. We used ARTIC V3 primers (Integrated DNA Technologies) for SARS-CoV-2 genome amplification and the Q5 High-Fidelity DNA Polymerase (New England Biolabs) in 2 multiplex PCR reactions. At this point we added a positive control from a previously sequenced sample and carried it throughout the protocol. We used the NEBNext Ultra II End repair/dA-tailing Module (New England Biolabs) to pool and dilute both reactions for the end prep reaction. We used Blunt/TA Ligase Master Mix (New England Biolabs) to ligate unique barcodes by using the Native Barcoding Expansion Kit 96 (Oxford Nanopore Technologies) to the end prepped samples. We pooled barcoded samples, which we cleaned up by using 0.4× volume of AMPure XP beads (Beckman Coulter, https://www.beckmancoulter.com) and quantified results by using the Qubit HS dsDNA kit (Thermo Fisher Scientific Inc.). We ligated sequencing adapters from Adapter Mix II Expansion Kit (Oxford Nanopore Technologies) by using the NEBNext Quick Ligation Module (New England Biolabs) and washed with Short Fragment Buffer (Oxford Nanopore Technologies). We eluted the final library on Elution Buffer (Oxford Nanopore Technologies) and quantified using the Qubit HS dsDNA kit. Approximately 50 fmol was loaded into a FLO-MIN106D R9.4.1 flow

cell (Oxford Nanopore Technologies) and sequenced on the GridION sequencing platform (Oxford Nanopore Technologies) until we achieved a minimum sequencing depth of 500×.

We performed basecalling and demultiplexing by using Guppy version 4.3.2 (Oxford Nanopore Technologies) in the high accuracy mode and both front and rear barcodes and the require_both_barcodes option were used for demultiplexed. We generated consensus genomes and used the poreCov pipeline (*4*; C. Brandt et al., unpub. data, https://doi.org/10.1101/2021.05.07.443089) implemented in Nextflow (*5*), which includes the ARTIC Network workflow (*6*), PANGOLIN (*7*) for lineage assignment, nextclade (https://github.com/nextstrain/nextclade) for clade assignment, Kraken2 and Krona (*8*,*9*) for contamination detection and visualization, minimap2 (*10*) for read mapping, medaka (https://nanoporetech.github.io/medaka) for consensus generation, and we used singularity as container engine (*11*). We did not include amplicons that were not sequenced or with a depth <20× in the consensus sequences; these positions are represented by stretches of N (uncalled bases). We kept complete sequences with ≤15% Ns for further analysis. We uploaded all genomes obtained in this study to the EpiCoV database in the GISAID initiative under the accession numbers EPI_ISL_1992241, EPI_ISL_1992100, EPI_ISL_1991974, and EPI_ISL_20317–62 (Appendix 1 Table 1).

Phylogenetic and Phylogeographic Analysis

We manually curated 59 P.1 genome sequences from Uruguay in specific genome positions of interest, namely P.1 synapomorphies, and analyzed these in the context of additional P.1 sequences from South America downloaded from EpiCoV database (https://www.epicov.org) in the GISAID initiative (*12*; Appendix 2, https://wwwnc.cdc.gov/EID/article/27/11/21-1198-App2.pdf). We downloaded 905 high quality, complete sequences from EpiCoV that included full collection date information for samples collected before March 31, 2021. We performed alignment by using MAFFT version 7.471 (*13*). After careful alignment inspection, we excluded several sequences that were missing the characteristic insertions/deletions of P.1, including 11288–11296 deletion and 28269–28273 insertion. The remaining 691 sequences were from Brazil (n = 590), Chile (n = 72), Colombia (n = 4), French Guiana (n = 15), Peru (n = 2), and Suriname (n = 8) (Appendix 1 Table 2).

We conducted maximum-likelihood phylogenetic analysis of P.1 samples by using IQ-TREE version 1.6.12 (http://www.iqtree.org) under the GTR+F+R3 nucleotide substitution model selected by the built-in ModelFinder option (*14*). We assessed branch support by the approximate likelihood-ratio test according to Shimodaira-Hasegawa–like procedure with 1,000 replicates. We used GISAID sequence EPI_ISL_833137 collected on December 4, 2020 as the tree root and visualized the tree by using iTOL (*15*). We then performed maximum-likelihood phylogeographic analysis to infer the geographic source of P.1 samples in Uruguay and time-scaled Bayesian analysis to estimate the time of the most recent common ancestors (T_{MRCA}) of the P.1 clades in Uruguay. We used TreeTime (*16*) to time-scaled the previously generated maximum-likelihood phylogenetic tree applying a fixed clock rate of 8×10^{-4} substitutions/site/year, based on previous estimates (*17*,*18*), a skyline coalescent model with 8 grid points and keeping polytomies. The time-scaled tree was then used for the ancestral character state reconstruction of epidemic locations with PastML (*19*), by using the marginal posterior probabilities approximation method with an F81-like model. We constructed time-scaled Bayesian phylogenetic tree by using the Bayesian Markov chain Monte Carlo approach implemented in BEAST version 1.10 (*20*) with BEAGLE library version 3 (http://beagle-lib.googlecode.com) to improve computational time. We conducted Bayesian analysis by using the nonparametric Bayesian skyline model as the coalescent tree prior (*21*), the GTR+I+G model of nucleotide substitution and a strict molecular clock model with a uniform prior on substitution rate of $8–10 \times 10^{-4}$ substitutions/site/year. We ran 2 Markov chains for 50 million generations and then combined the 2 to ensure stationarity and good mixing. To assess convergence (effective sample size >200) in parameter estimates by using TRACER version 1.7 (*22*). We summarized the maximum clade credibility tree by using TreeAnnotator version 1.10 (*23*) and the tree was visualized with FigTree version 1.4.4 (http://tree.bio.ed.ac.uk/software/figtree).

References

1. Integrated DNA Technologies. OligoAnalyzer tool [cited 2021 Feb 15]. https://www.idtdna.com/pages/tools/oligoanalyzer

2. Vogels CBF, Breban MI, et al. PCR assay to enhance global surveillance for SARS-CoV-2 variants of concern. PLoS Biol. 2021;19:e3001236. PubMed https://doi.org/10.1371/journal.pbio.3001236

3. Quick J. nCoV-2019 sequencing protocol V.3 (LoCost). protocols.io [cited 2021 Apr 30]. https://www.protocols.io/view/ncov-2019-sequencing-protocol-v3-locost-bh42j8ye

4. Hufsky F, Lamkiewicz K, Almeida A, Aouacheria A, Arighi C, Bateman A, et al. Computational strategies to combat COVID-19: useful tools to accelerate SARS-CoV-2 and coronavirus research. Brief Bioinform. 2021;22:642–63. PubMed https://doi.org/10.1093/bib/bbaa232

5. Di Tommaso P, Chatzou M, Floden EW, Barja PP, Palumbo E, Notredame C. Nextflow enables reproducible computational workflows. Nat Biotechnol. 2017;35:316–9. PubMed https://doi.org/10.1038/nbt.3820

6. Loman N, Rowe W, Rambaut A. nCoV-2019 novel coronavirus bioinformatics protocol [cited 2021 Apr 30]. https://artic.network/ncov-2019/ncov2019-bioinformatics-sop.html

7. Rambaut A, Holmes EC, O'Toole Á, Hill V, McCrone JT, Ruis C, et al. A dynamic nomenclature proposal for SARS-CoV-2 lineages to assist genomic epidemiology. Nat Microbiol. 2020;5:1403–7. PubMed https://doi.org/10.1038/s41564-020-0770-5

8. Wood DE, Lu J, Langmead B. Improved metagenomic analysis with Kraken 2. Genome Biol. 2019;20:257. PubMed https://doi.org/10.1186/s13059-019-1891-0

9. Ondov BD, Bergman NH, Phillippy AM. Interactive metagenomic visualization in a web browser. BMC Bioinformatics. 2011;12:385. PubMed https://doi.org/10.1186/1471-2105-12-385

10. Li H. Minimap2: pairwise alignment for nucleotide sequences. Bioinformatics. 2018;34:3094–100. PubMed https://doi.org/10.1093/bioinformatics/bty191

11. Kurtzer GM, Sochat V, Bauer MW. Singularity: scientific containers for mobility of compute. PLoS One. 2017;12:e0177459. PubMed https://doi.org/10.1371/journal.pone.0177459

12. Shu Y, McCauley J. GISAID: Global initiative on sharing all influenza data - from vision to reality. Euro Surveill. 2017;22:30494. PubMed https://doi.org/10.2807/1560-7917.ES.2017.22.13.30494

13. Katoh K, Standley DM. MAFFT multiple sequence alignment software version 7: improvements in performance and usability. Mol Biol Evol. 2013;30:772–80. PubMed https://doi.org/10.1093/molbev/mst010

14. Nguyen LT, Schmidt HA, von Haeseler A, Minh BQ. IQ-TREE: a fast and effective stochastic algorithm for estimating maximum-likelihood phylogenies. Mol Biol Evol. 2015;32:268–74. PubMed https://doi.org/10.1093/molbev/msu300

15. Letunic I, Bork P. Interactive Tree Of Life (iTOL) v4: recent updates and new developments. Nucleic Acids Res. 2019;47:W256–9. PubMed https://doi.org/10.1093/nar/gkz239

16. Sagulenko P, Puller V, Neher RA. TreeTime: Maximum-likelihood phylodynamic analysis. Virus Evol. 2018;4:vex042. PubMed https://doi.org/10.1093/ve/vex042

17. Duchene S, Featherstone L, Haritopoulou-Sinanidou M, Rambaut A, Lemey P, Baele G. Temporal signal and the phylodynamic threshold of SARS-CoV-2. Virus Evol. 2020;6:veaa061. PubMed https://doi.org/10.1093/ve/veaa061

18. Ghafari V, du Plessis L, Pybus O, Katzourakis A. Time dependence of SARS-CoV-2 substitution rates [cited 2021 Apr 10]. https://virological.org/t/time-dependence-of-sars-cov-2-substitution-rates/542

19. Ishikawa SA, Zhukova A, Iwasaki W, Gascuel O. A fast likelihood method to reconstruct and visualize ancestral scenarios. Mol Biol Evol. 2019;36:2069–85. PubMed https://doi.org/10.1093/molbev/msz131

20. Suchard MA, Lemey P, Baele G, Ayres DL, Drummond AJ, Rambaut A. Bayesian phylogenetic and phylodynamic data integration using BEAST 1.10. Virus Evol. 2018;4:vey016. PubMed https://doi.org/10.1093/ve/vey016

21. Drummond AJ, Rambaut A, Shapiro B, Pybus OG. Bayesian coalescent inference of past population dynamics from molecular sequences. Mol Biol Evol. 2005;22:1185–92. PubMed https://doi.org/10.1093/molbev/msi103

22. Rambaut A, Drummond AJ, Xie D, Baele G, Suchard MA. Posterior summarization in Bayesian phylogenetics using Tracer 1.7. Syst Biol. 2018;67:901–4. PubMed https://doi.org/10.1093/sysbio/syy032

23. Bouckaert R, Vaughan TG, Barido-Sottani J, Duchêne S, Fourment M, Gavryushkina A, et al. BEAST 2.5: an advanced software platform for Bayesian evolutionary analysis. PLoS Comput Biol. 2019;15:e1006650. PubMed https://doi.org/10.1371/journal.pcbi.1006650

Appendix 1 Table 1. Information on 251 SARS-CoV-2–positive nasopharyngeal swab samples analyzed by PCR from departments, Uruguay*

Sample identification	Cycle threshold	Department	Date collected	Variants by PCR
CUY1–000001	20.1	Treinta y Tres	2021 Mar 4	No VOC
CUY1–000002	23.8	Treinta y Tres	2021 Mar 4	No VOC
CUY1–000004	28.7	Artigas	2021 Mar 4	No VOC
CUY1–000006	18.0	Artigas	2021 Mar 4	No VOC
CUY1–000007	21.5	San José	2021 Mar 4	No VOC
CUY1–000008	22.7	San José	2021 Mar 4	No VOC
CUY1–000011	22.8	Artigas	2021 Mar 4	**P.1/B.1.351**
CUY1–000012	23.0	Artigas	2021 Mar 4	No VOC
CUY1–000015	23.5	Artigas	2021 Mar 4	No VOC
CUY1–000017	29.7	Montevideo	2021 Mar 4	No VOC
CUY1–000020	34.7	Montevideo	2021 Mar 4	**P.1/B.1.351**
CUY1–000021	22.9	Colonia	2021 Mar 4	No VOC
CUY1–000022	19.6	Maldonado	2021 Mar 4	No VOC
CUY1–000023	18.3	Maldonado	2021 Mar 4	No VOC
CUY1–000024	26.9	Montevideo	2021 Mar 4	No VOC
CUY1–000025	15.4	Montevideo	2021 Mar 5	**P.1/B.1.351**
CUY1–000028	31.5	Salto	2021 Mar 5	No VOC
CUY1–000029	18.7	Salto	2021 Mar 5	No VOC
CUY1–000030	28.7	Colonia	2021 Mar 5	NC
CUY1–000031	21.3	Artigas	2021 Mar 5	**P.1/B.1.351**
CUY1–000032	22.9	San José	2021 Mar 5	No VOC
CUY1–000034	29.5	Canelones	2021 Mar 5	No VOC
CUY1–000035	27.9	Canelones	2021 Mar 5	No VOC
CUY1–000036	29.1	Salto	2021 Mar 5	No VOC
CUY1–000037	29.9	Flores	2021 Mar 5	No VOC
CUY1–000038	25.4	Salto	2021 Mar 5	No VOC
CUY1–000039	24.4	Flores	2021 Mar 5	No VOC
CUY1–000040	27.9	Tacuarembó	2021 Mar 4	No VOC
CUY1–000041	30.1	Flores	2021 Mar 5	No VOC
CUY1–000042	19.0	Canelones	2021 Mar 5	No VOC
CUY1–000043	29.6	Canelones	2021 Mar 5	No VOC
CUY1–000045	20.8	ND	2021 Mar 5	**P.1/B.1.351**
CUY1–000046	25.9	Canelones	2021 Mar 5	**P.1/B.1.351**
CUY1–000047	25.5	Canelones	2021 Mar 5	**P.1/B.1.351**
CUY1–000048	22.4	Salto	2021 Mar 5	No VOC
CUY1–000049	20.2	San José	2021 Mar 5	**P.1/B.1.351**
CUY1–000050	22.7	San José	2021 Mar 5	**P.1/B.1.351**
CUY1–000051	24.9	Salto	2021 Mar 5	No VOC
CUY1–000052	25.2	Salto	2021 Mar 5	No VOC
CUY1–000053	33.0	Tacuarembó	2021 Mar 5	No VOC
CUY1–000054	27.5	Salto	2021 Mar 5	No VOC
CUY1–000055	20.9	Salto	2021 Mar 5	No VOC
CUY1–000056	18.7	Canelones	2021 Mar 5	No VOC
CUY1–000057	21.8	Tacuarembó	2021 Mar 5	No VOC
CUY1–000060	22.8	Florida	2021 Mar 11	No VOC
CUY1–000061	16.4	Rocha	2021 Mar 11	No VOC
CUY1–000062	18.2	Rocha	2021 Mar 12	**P.1/B.1.351**
CUY1–000063	20.0	Florida	2021 Mar 12	No VOC
CUY1–000064	21.9	Río Negro	2021 Mar 12	No VOC
CUY1–000065	20.2	Rocha	2021 Mar 12	No VOC
CUY1–000066	22.4	Rocha	2021 Mar 12	No VOC
CUY1–000067	22.1	Rocha	2021 Mar 12	No VOC
CUY1–000068	31.8	Flores	2021 Mar 12	No VOC
CUY1–000069	25.5	Flores	2021 Mar 12	No VOC
CUY1–000070	30.5	Flores	2021 Mar 12	P.1/B.1.351
CUY1–000071	34.1	Río Negro	2021 Mar 12	P.1/B.1.351
CUY1–000072	25.9	Río Negro	2021 Mar 12	**P.1/B.1.351**
CUY1–000073	22.2	Flores	2021 Mar 12	No VOC
CUY1–000074	ND	ND	2021 Mar 12	No VOC
CUY1–000075	16.4	San José	2021 Mar 12	**P.1/B.1.351**
CUY1–000076	19.2	Canelones	2021 Mar 12	No VOC
CUY1–000077	20.9	San José	2021 Mar 12	No VOC
CUY1–000078	26.8	Salto	2021 Mar 12	No VOC
CUY1–000079	26.3	Salto	2021 Mar 12	No VOC
CUY1–000080	19.9	Río Negro	2021 Mar 12	**P.1/B.1.351**
CUY1–000081	29.5	Artigas	2021 Mar 12	P.1/B.1.351
CUY1–000082	19.2	Artigas	2021 Mar 12	No VOC

Sample identification	Cycle threshold	Department	Date collected	Variants by PCR
CUY1–000083	17.2	Artigas	2021 Mar 12	No VOC
CUY1–000084	25.5	ND	2021 Mar 12	P.1/B.1.351
CUY1–000085	18.1	Salto	2021 Mar 12	No VOC
CUY1–000086	19.4	Colonia	2021 Mar 13	No VOC
CUY1–000087	26.6	Florida	2021 Mar 13	No VOC
CUY1–000088	23.9	Flores	2021 Mar 13	No VOC
CUY1–000089	18.6	San José	2021 Mar 13	No VOC
CUY1–000090	21.4	San José	2021 Mar 13	**P.1/B.1.351**
CUY1–000091	22.2	Montevideo	2021 Mar 13	No VOC
CUY1–000092	20.1	Canelones	2021 Mar 13	No VOC
CUY1–000093	19.0	Canelones	2021 Mar 13	**P.1/B.1.351**
CUY1–000094	17.6	Canelones	2021 Mar 13	**P.1/B.1.351**
CUY1–000095	23.0	San José	2021 Mar 13	**P.1/B.1.351**
CUY1–000096	19.9	Montevideo	2021 Mar 14	No VOC
CUY1–000097	29.2	Montevideo	2021 Mar 14	**P.1/B.1.351**
CUY1–000098	24.5	ND	ND	No VOC
CUY1–000099	29.7	Canelones	2021 Mar 12	No VOC
CUY1–000100	15.0	Salto	2021 Feb 24	No VOC
CUY1–000101	11.0	Salto	2021 Feb 25	No VOC
CUY1–000102	9.0	Salto	2021 Feb 26	No VOC
CUY1–000103	13.0	Salto	2021 Mar 1	No VOC
CUY1–000104	15.0	Salto	2021 Mar 1	No VOC
CUY1–000105	11.0	Salto	2021 Mar 1	No VOC
CUY1–000106	12.0	Salto	2021 Mar 1	No VOC
CUY1–000107	15.0	Río Negro	2021 Mar 2	No VOC
CUY1–000108	12.0	Salto	2021 Mar 2	No VOC
CUY1–000109	12.0	Salto	2021 Mar 2	No VOC
CUY1–000110	13.0	Río Negro	2021 Mar 3	**P.1/B.1.351**
CUY1–000111	13.0	Salto	2021 Mar 4	No VOC
CUY1–000112	14.0	Salto	2021 Mar 4	No VOC
CUY1–000113	12.0	Salto	2021 Mar 8	No VOC
CUY1–000114	10.0	Río Negro	2021 Mar 8	**P.1/B.1.351**
CUY1–000115	17.0	Río Negro	2021 Mar 8	**P.1/B.1.351**
CUY1–000116	14.0	Salto	2021 Mar 9	No VOC
CUY1–000117	14.0	Salto	2021 Mar 9	No VOC
CUY1–000118	17.0	Salto	2021 Mar 9	No VOC
CUY1–000119	10.0	Salto	2021 Mar 9	No VOC
CUY1–000120	18.0	Salto	2021 Mar 10	No VOC
CUY1–000121	12.0	Salto	2021 Mar 10	No VOC
CUY1–000122	13.0	Salto	2021 Mar 10	No VOC
CUY1–000123	12.0	Salto	2021 Mar 10	No VOC
CUY1–000124	17.0	Salto	2021 Mar 10	No VOC
CUY1–000125	13.0	Salto	2021 Mar 11	**P.1/B.1.351**
CUY1–000126	16.0	Salto	2021 Mar 11	No VOC
CUY1–000127	18.0	Salto	2021 Mar 11	No VOC
CUY1–000128	16.0	Salto	2021 Mar 11	No VOC
CUY1–000129	11.0	Salto	2021 Mar 11	**P.1/B.1.351**
CUY1–000130	18.0	Tacuarembó	2020 Nov 13	No VOC
CUY1–000131	15.0	Tacuarembó	2020 Nov 13	No VOC
CUY1–000132	17.0	Tacuarembó	2020 Nov 13	No VOC
CUY1–000133	19.0	Tacuarembó	2020 Nov 16	No VOC
CUY1–000134	17.0	Tacuarembó	2020 Nov 16	No VOC
CUY1–000135	16.0	Tacuarembó	2020 Nov 16	No VOC
CUY1–000136	19.0	Tacuarembó	2020 Nov 16	No VOC
CUY1–000137	15.0	Tacuarembó	2020 Nov 16	No VOC
CUY1–000138	18.0	Tacuarembó	2020 Nov 23	No VOC
CUY1–000139	14.0	Tacuarembó	2020 Dec 1	No VOC
CUY1–000140	19.0	Tacuarembó	2020 Dec 1	No VOC
CUY1–000141	13.0	Tacuarembó	2020 Dec 1	No VOC
CUY1–000142	19.0	Tacuarembó	2020 Dec 4	No VOC
CUY1–000143	18.6	Tacuarembó	2020 Dec 8	No VOC
CUY1–000144	18.0	Tacuarembó	2020 Dec 15	No VOC
CUY1–000145	18.2	Tacuarembó	2020 Dec 18	No VOC
CUY1–000146	14.2	Tacuarembó	2020 Dec 21	No VOC
CUY1–000147	15.7	Tacuarembó	2020 Dec 21	No VOC
CUY1–000148	18.0	Tacuarembó	2020 Dec 22	No VOC
CUY1–000149	18.5	Tacuarembó	2020 Dec 22	No VOC
CUY1–000150	15.2	Tacuarembó	2020 Dec 23	No VOC
CUY1–000151	18.0	Tacuarembó	2020 Dec 23	No VOC

Sample identification	Cycle threshold	Department	Date collected	Variants by PCR
CUY1–000152	16.3	Tacuarembó	2020 Dec 24	No VOC
CUY1–000153	18.5	Tacuarembó	2020 Dec 24	No VOC
CUY1–000154	18.5	Tacuarembó	2020 Dec 24	No VOC
CUY1–000155	19.2	Tacuarembó	2020 Dec 24	No VOC
CUY1–000156	18.1	Tacuarembó	2020 Dec 24	No VOC
CUY1–000157	19.5	Tacuarembó	2020 Dec 24	No VOC
CUY1–000158	19.8	Tacuarembó	2021 Jan 3	No VOC
CUY1–000159	18.7	Tacuarembó	2021 Jan 3	No VOC
CUY1–000160	19.4	Tacuarembó	2021 Jan 5	No VOC
CUY1–000161	15.2	Tacuarembó	2021 Jan 6	No VOC
CUY1–000162	11.8	Tacuarembó	2021 Jan 6	No VOC
CUY1–000163	17.5	Tacuarembó	2021 Jan 6	No VOC
CUY1–000164	17.3	Tacuarembó	2021 Jan 7	No VOC
CUY1–000165	14.1	Tacuarembó	2021 Jan 7	No VOC
CUY1–000166	17.3	Tacuarembó	2020 Nov 1	No VOC
CUY1–000167	15.3	Tacuarembó	2021 Jan 18	No VOC
CUY1–000168	15.3	Tacuarembó	2021 Jan 20	No VOC
CUY1–000169	14.5	Tacuarembó	2021 Jan 20	No VOC
CUY1–000170	15.5	Tacuarembó	2021 Jan 20	No VOC
CUY1–000171	13.2	Tacuarembó	2021 Jan 21	No VOC
CUY1–000172	13.4	Tacuarembó	2021 Jan 21	No VOC
CUY1–000173	17.9	Tacuarembó	2021 Jan 21	No VOC
CUY1–000174	16.5	Tacuarembó	2021 Jan 28	No VOC
CUY1–000175	17.3	Tacuarembó	2021 Jan 29	No VOC
CUY2–000176	14.0	Paysandú	2021 Jan 11	No VOC
CUY2–000177	13.0	Paysandú	2021 Jan 11	No VOC
CUY2–000178	12.0	Salto	2021 Jan 12	No VOC
CUY2–000179	14.0	Salto	2021 Jan 14	No VOC
CUY2–000180	17.0	Paysandú	2021 Jan 13	No VOC
CUY2–000181	16.0	Paysandú	2021 Jan 14	No VOC
CUY2–000182	14.0	Salto	2021 Jan 19	No VOC
CUY2–000183	12.0	Montevideo	2021 Jan 19	No VOC
CUY2–000184	16.0	Salto	2021 Jan 19	No VOC
CUY2–000185	17.0	Salto	2021 Jan 20	No VOC
CUY2–000186	9.0	Salto	2021 Jan 20	No VOC
CUY2–000187	9.0	Salto	2021 Jan 21	No VOC
CUY2–000188	12.0	Salto	2021 Jan 21	No VOC
CUY2–000189	14.0	Salto	2021 Jan 22	No VOC
CUY2–000190	13.0	Salto	2021 Jan 22	No VOC
CUY2–000191	16.0	Paysandú	2021 Feb 5	No VOC
CUY2–000192	16.0	Paysandú	2021 Feb 8	No VOC
CUY2–000193	14.0	Paysandú	2021 Feb 9	No VOC
CUY2–000194	16.0	Paysandú	2021 Feb 8	No VOC
CUY2–000195	11.0	Salto	2021 Mar 15	No VOC
CUY2–000196	12.0	Salto	2021 Mar 15	No VOC
CUY2–000197	11.0	Salto	2021 Mar 15	No VOC
CUY2–000198	13.0	Salto	2021 Mar 18	**P.1/B.1.351**
CUY2–000199	14.0	Río Negro	2021 Mar 18	**P.1/B.1.351**
CUY2–000200	12.0	Río Negro	2021 Mar 18	**P.1/B.1.351**
CUY2–000201	22.9	Montevideo	2021 Mar 12	No VOC
CUY2–000202	20.0	Montevideo	2021 Mar 23	No VOC
CUY2–000203	16.8	Montevideo	2021 Mar 23	**P.1/B.1.351**
CUY2–000204	13.6	Montevideo	2021 Mar 22	**P.1/B.1.351**
CUY2–000205	24.5	Montevideo	2021 Mar 19	No VOC
CUY2–000206	13.5	Montevideo	2021 Mar 22	No VOC
CUY2–000207	28.2	Montevideo	2021 Mar 22	No VOC
CUY2–000208	22.7	Tacuarembó	ND	No VOC
CUY2–000209	21.8	Tacuarembó	2021 Mar 18	No VOC
CUY2–000210	31.5	Artigas	2021 Mar 18	No VOC
CUY2–000211	22.3	Río Negro	2021 Mar 18	**P.1/B.1.351**
CUY2–000212	21.9	Río Negro	2021 Mar 18	**P.1/B.1.351**
CUY2–000213	20.3	Río Negro	2021 Mar 19	**P.1/B.1.351**
CUY2–000214	21.9	Río Negro	2021 Mar 18	**P.1/B.1.351**
CUY2–000215	23.6	Artigas	2021 Mar 19	**P.1/B.1.351**
CUY2–000216	28.8	Soriano	2021 Mar 19	P.1/B.1.351
CUY2–000217	21.0	San José	2021 Mar 19	No VOC
CUY2–000218	24.2	Soriano	2021 Mar 19	No VOC
CUY2–000219	22.7	Soriano	2021 Mar 19	No VOC
CUY2–000220	ND	ND	2021 Mar 19	No VOC

Sample identification	Cycle threshold	Department	Date collected	Variants by PCR
CUY2–000221	24.4	Rocha	2021 Mar 19	No VOC
CUY2–000222	22.8	San José	2021 Mar 19	**P.1/B.1.351**
CUY2–000223	25.9	San José	2021 Mar 19	No VOC
CUY2–000224	23.2	Maldonado	2021 Mar 19	No VOC
CUY2–000225	23.4	Canelones	2021 Mar 19	P.1/B.1.351
CUY2–000226	22.0	Canelones	2021 Mar 19	No VOC
CUY2–000227	23.0	Rocha	2021 Mar 19	No VOC
CUY2–000228	19.7	Rocha	2021 Mar 19	**P.1/B.1.351**
CUY2–000229	20.0	Rocha	2021 Mar 19	No VOC
CUY2–000231	18.1	Canelones	2021 Mar 19	**P.1/B.1.351**
CUY2–000232	24.4	Soriano	2021 Mar 19	No VOC
CUY2–000233	21.3	Rocha	2021 Mar 19	**P.1/B.1.351**
CUY2–000234	20.5	Canelones	2021 Mar 19	No VOC
CUY2–000235	19.9	Río Negro	2021 Mar 19	**P.1/B.1.351**
CUY2–000236	19.2	Río Negro	2021 Mar 19	**P.1/B.1.351**
CUY2–000237	19.6	Río Negro	2021 Mar 19	**P.1/B.1.351**
CUY2–000238	23.6	Río Negro	2021 Mar 19	**P.1/B.1.351**
CUY2–000239	26.2	Río Negro	2021 Mar 19	**P.1/B.1.351**
CUY2–000240	12.3	Río Negro	2021 Mar 19	**P.1/B.1.351**
CUY2–000241	22.8	Canelones	2021 Mar 20	No VOC
CUY2–000242	19.5	Canelones	2021 Mar 20	No VOC
CUY2–000243	24.7	Canelones	2021 Mar 20	No VOC
CUY2–000244	25.9	Canelones	2021 Mar 20	**P.1/B.1.351**
CUY2–000245	18.0	Canelones	2021 Mar 20	**P.1/B.1.351**
CUY2–000246	30.3	Canelones	2021 Mar 20	P.1/B.1.351
CUY2–000247	21.5	San José	2021 Mar 20	No VOC
CUY2–000249	23.5	Canelones	2021 Mar 20	No VOC
CUY2–000250	21.6	Artigas	2021 Mar 20	**P.1/B.1.351**
CUY2–000251	31.2	Montevideo	2021 Mar 20	No VOC
CUY2–000252	17.2	Montevideo	2021 Mar 20	**P.1/B.1.351**
CUY2–000253	23.3	Montevideo	2021 Mar 20	No VOC
CUY2–000254	20.6	Montevideo	2021 Mar 20	**P.1/B.1.351**
CUY2–000255	24.6	Montevideo	2021 Mar 20	No VOC
CUY2–000256	19.5	Montevideo	2021 Mar 20	**P.1/B.1.351**
CUY2–000257	23.2	Canelones	2021 Mar 20	**P.1/B.1.351**
CUY2–000258	17.7	Montevideo	2021 Mar 21	**P.1/B.1.351**
CUY2–000259	26.8	Soriano	2021 Mar 21	**P.1/B.1.351**
CUY2–000260	25.3	Soriano	2021 Mar 21	**P.1/B.1.351**
CUY2–000261	26.7	Colonia	2021 Mar 22	**P.1/B.1.351**
CUY2–000262	22.7	Montevideo	2021 Mar 26	P.1/B.1.351
CUY2–000263	18.3	Montevideo	2021 Mar 26	**P.1/B.1.351**
CUY2–000264	23.6	Montevideo	2021 Mar 26	P.1/B.1.351
CUY2–000265	20.7	Montevideo	2021 Mar 26	**P.1/B.1.351**
CUY2–000266	18.3	Montevideo	2021 Mar 26	No VOC
CUY2–000267	18.3	Tacuarembó	2021 Mar 21	P.1/B.1.351
CUY2–000268	20.1	Tacuarembó	2021 Mar 26	**P.1/B.1.351**
M525_UYRO	19.0	Rocha	2021 Feb 26	P.1/B.1.351
M530_UYRO	14.6	Rocha	2021 Feb 23	ND
M531_UYRO	12.3	Rocha	2021 Feb 23	ND

*Bold text indicates samples that were successfully sequenced. NC, not conclusive by PCR VOC assay; ND, no data; VOC, variant of concern; SARS-CoV-2, severe acute respiratory syndrome coronavirus 2.

Appendix 1 Table 2. EpiCoV database identifiers (GISAID initiative) for SARS-CoV-2 downloaded sequences used to assess variants of concern, Uruguay*

Sample identification	Date collected	Date uploaded	Collection locations
EPI_ISL_1001384	2021 Jan 31	2021 Feb 15	South America / French Guiana / Cayenne
EPI_ISL_1001385	2021 Jan 31	2021 Feb 15	South America / French Guiana / Cayenne
EPI_ISL_1034304	2021 Jan 19	2021 Feb 19	South America / Brazil / Amazonas / Manaus
EPI_ISL_1034306	2021 Jan 29	2021 Feb 19	South America / Brazil / Amazonas / Manaus
EPI_ISL_1039691	2021 Jan 19	2021 Feb 20	South America / Brazil / Goias / Aparecida de Goiania
EPI_ISL_1039692	2021 Jan 19	2021 Feb 20	South America / Brazil / Goias / Aparecida de Goiania
EPI_ISL_1039693	2021 Jan 19	2021 Feb 20	South America / Brazil / Goias / Goiania
EPI_ISL_1039694	2021 Jan 19	2021 Feb 20	South America / Brazil / Goias / Goiania
EPI_ISL_1039695	2021 Jan 19	2021 Feb 20	South America / Brazil / Goias / Goiania
EPI_ISL_1041509	2021 Jan 20	2021 Feb 22	South America / Brazil / Goias / Goiania
EPI_ISL_1068110	2020 Dec 28	2021 Feb 25	South America / Brazil / Amazonas / Manaus
EPI_ISL_1068111	2020 Dec 28	2021 Feb 25	South America / Brazil / Amazonas / Manaus
EPI_ISL_1068112	2020 Dec 29	2021 Feb 25	South America / Brazil / Amazonas / Manaus
EPI_ISL_1068114	2020 Dec 23	2021 Feb 25	South America / Brazil / Amazonas / Manaus
EPI_ISL_1068149	2020 Dec 18	2021 Feb 25	South America / Brazil / Amazonas / Manaus
EPI_ISL_1068150	2020 Dec 18	2021 Feb 25	South America / Brazil / Amazonas / Manaus
EPI_ISL_1068151	2020 Dec 21	2021 Feb 25	South America / Brazil / Amazonas / Manaus
EPI_ISL_1068154	2020 Dec 23	2021 Feb 25	South America / Brazil / Amazonas / Manaus
EPI_ISL_1068156	2020 Dec 27	2021 Feb 25	South America / Brazil / Amazonas / Manaus
EPI_ISL_1068157	2020 Dec 28	2021 Feb 25	South America / Brazil / Amazonas / Manaus
EPI_ISL_1068158	2020 Dec 28	2021 Feb 25	South America / Brazil / Amazonas / Manaus
EPI_ISL_1068159	2020 Dec 28	2021 Feb 25	South America / Brazil / Amazonas / Manaus
EPI_ISL_1068160	2020 Dec 29	2021 Feb 25	South America / Brazil / Amazonas / Manaus
EPI_ISL_1068169	2020 Dec 28	2021 Feb 25	South America / Brazil / Amazonas / Rio Preto da Eva
EPI_ISL_1068198	2020 Dec 24	2021 Feb 25	South America / Brazil / Amazonas / Manaus
EPI_ISL_1068221	2020 Dec 21	2021 Feb 25	South America / Brazil / Amazonas / Manaus
EPI_ISL_1068222	2020 Dec 21	2021 Feb 25	South America / Brazil / Amazonas / Manaus
EPI_ISL_1068225	2020 Dec 23	2021 Feb 25	South America / Brazil / Amazonas / Manaus
EPI_ISL_1068226	2020 Dec 23	2021 Feb 25	South America / Brazil / Amazonas / Manaus
EPI_ISL_1068243	2020 Dec 23	2021 Feb 25	South America / Brazil / Amazonas / Manaus
EPI_ISL_1068248	2020 Dec 28	2021 Feb 25	South America / Brazil / Amazonas / Careiro
EPI_ISL_1068249	2020 Dec 22	2021 Feb 25	South America / Brazil / Amazonas / Manaus
EPI_ISL_1068258	2021 Jan 13	2021 Feb 25	South America / Brazil / Amazonas / Manaus
EPI_ISL_1068260	2021 Jan 4	2021 Feb 25	South America / Brazil / Amazonas / Manaus
EPI_ISL_1068261	2021 Jan 5	2021 Feb 25	South America / Brazil / Amazonas / Manaus
EPI_ISL_1068262	2021 Jan 5	2021 Feb 25	South America / Brazil / Amazonas / Manaus
EPI_ISL_1068263	2021 Jan 6	2021 Feb 25	South America / Brazil / Amazonas / Manaus
EPI_ISL_1068264	2021 Jan 6	2021 Feb 25	South America / Brazil / Amazonas / Manaus
EPI_ISL_1068266	2021 Jan 7	2021 Feb 25	South America / Brazil / Amazonas / Manaus
EPI_ISL_1068268	2021 Jan 8	2021 Feb 25	South America / Brazil / Amazonas / Manaus
EPI_ISL_1068269	2021 Jan 8	2021 Feb 25	South America / Brazil / Amazonas / Manaus
EPI_ISL_1068270	2021 Jan 11	2021 Feb 25	South America / Brazil / Amazonas / Manaus
EPI_ISL_1068271	2021 Jan 11	2021 Feb 25	South America / Brazil / Amazonas / Manaus
EPI_ISL_1068272	2021 Jan 12	2021 Feb 25	South America / Brazil / Amazonas / Manaus
EPI_ISL_1068273	2021 Jan 12	2021 Feb 25	South America / Brazil / Amazonas / Manaus
EPI_ISL_1068274	2021 Jan 8	2021 Feb 25	South America / Brazil / Amazonas / Manaus
EPI_ISL_1068275	2021 Jan 8	2021 Feb 25	South America / Brazil / Amazonas / Manaus
EPI_ISL_1068276	2021 Jan 13	2021 Feb 25	South America / Brazil / Amazonas / Manaus
EPI_ISL_1068278	2021 Jan 13	2021 Feb 25	South America / Brazil / Amazonas / Manaus
EPI_ISL_1068279	2021 Jan 7	2021 Feb 25	South America / Brazil / Amazonas / Manaus
EPI_ISL_1068280	2021 Jan 6	2021 Feb 25	South America / Brazil / Amazonas / Manaus
EPI_ISL_1068281	2021 Jan 6	2021 Feb 25	South America / Brazil / Amazonas / Manaus
EPI_ISL_1068282	2021 Jan 11	2021 Feb 25	South America / Brazil / Amazonas / Manaus
EPI_ISL_1068283	2021 Jan 12	2021 Feb 25	South America / Brazil / Amazonas / Manaus
EPI_ISL_1068284	2021 Jan 13	2021 Feb 25	South America / Brazil / Amazonas / Manaus
EPI_ISL_1068285	2021 Jan 4	2021 Feb 25	South America / Brazil / Amazonas / Anori
EPI_ISL_1068286	2021 Jan 4	2021 Feb 25	South America / Brazil / Amazonas / São Gabriel da Cachoeira
EPI_ISL_1068287	2021 Jan 5	2021 Feb 25	South America / Brazil / Amazonas / Iranduba
EPI_ISL_1068288	2021 Jan 5	2021 Feb 25	South America / Brazil / Amazonas / Rio Preto da Eva
EPI_ISL_1068289	2021 Jan 5	2021 Feb 25	South America / Brazil / Amazonas / Presidente Figueiredo
EPI_ISL_1068290	2021 Jan 5	2021 Feb 25	South America / Brazil / Amazonas / Tabatinga
EPI_ISL_1068291	2021 Jan 7	2021 Feb 25	South America / Brazil / Amazonas / Careiro
EPI_ISL_1068292	2021 Jan 6	2021 Feb 25	South America / Brazil / Amazonas / Manacapuru
EPI_ISL_1078992	2021 Jan 15	2021 Feb 25	South America / Brazil / Sao Paulo / Bauru
EPI_ISL_1079002	2021 Feb 5	2021 Feb 25	South America / Brazil / Sao Paulo / Bauru
EPI_ISL_1079008	2021 Feb 11	2021 Feb 25	South America / Brazil / Sao Paulo / Lencois Paulista

Sample identification	Date collected	Date uploaded	Collection locations
EPI_ISL_1079162	2021 Feb 8	2021 Feb 25	South America / Brazil / Sao Paulo / Bauru
EPI_ISL_1086034	2021 Feb 11	2021 Feb 26	South America / Brazil / Sao Paulo / Araraquara
EPI_ISL_1086035	2021 Feb 10	2021 Feb 26	South America / Brazil / Sao Paulo / Lencois Paulista
EPI_ISL_1086036	2021 Feb 10	2021 Feb 26	South America / Brazil / Sao Paulo / Lencois Paulista
EPI_ISL_1086037	2021 Feb 9	2021 Feb 26	South America / Brazil / Sao Paulo / Araraquara
EPI_ISL_1086038	2021 Feb 9	2021 Feb 26	South America / Brazil / Sao Paulo / Araraquara
EPI_ISL_1086039	2021 Feb 9	2021 Feb 26	South America / Brazil / Sao Paulo / Araraquara
EPI_ISL_1086040	2021 Feb 9	2021 Feb 26	South America / Brazil / Sao Paulo / Araraquara
EPI_ISL_1086041	2021 Feb 9	2021 Feb 26	South America / Brazil / Sao Paulo / Araraquara
EPI_ISL_1086042	2021 Feb 9	2021 Feb 26	South America / Brazil / Sao Paulo / Araraquara
EPI_ISL_1086043	2021 Feb 9	2021 Feb 26	South America / Brazil / Sao Paulo / Araraquara
EPI_ISL_1086044	2021 Feb 8	2021 Feb 26	South America / Brazil / Sao Paulo / Lencois Paulista
EPI_ISL_1086045	2021 Feb 5	2021 Feb 26	South America / Brazil / Sao Paulo / Lencois Paulista
EPI_ISL_1086046	2021 Feb 4	2021 Feb 26	South America / Brazil / Sao Paulo / Lencois Paulista
EPI_ISL_1086047	2021 Jan 30	2021 Feb 26	South America / Brazil / Sao Paulo / Jau
EPI_ISL_1086048	2021 Jan 14	2021 Feb 26	South America / Brazil / Sao Paulo / Jau
EPI_ISL_1086049	2021 Jan 11	2021 Feb 26	South America / Brazil / Sao Paulo / Jau
EPI_ISL_1096120	2021 Jan 15	2021 Feb 28	South America / Brazil / Sao Paulo / Jau
EPI_ISL_1096121	2021 Jan 25	2021 Feb 28	South America / Brazil / Sao Paulo / Araraquara
EPI_ISL_1096122	2021 Jan 26	2021 Feb 28	South America / Brazil / Sao Paulo / Jau
EPI_ISL_1096124	2021 Jan 28	2021 Feb 28	South America / Brazil / Sao Paulo / Dois Corregos
EPI_ISL_1096125	2021 Feb 2	2021 Feb 28	South America / Brazil / Sao Paulo / Jau
EPI_ISL_1096127	2021 Feb 3	2021 Feb 28	South America / Brazil / Sao Paulo / Bocaina
EPI_ISL_1096131	2021 Feb 8	2021 Feb 28	South America / Brazil / Sao Paulo / Lins
EPI_ISL_1096132	2021 Feb 8	2021 Feb 28	South America / Brazil / Sao Paulo / Lins
EPI_ISL_1096134	2021 Feb 8	2021 Feb 28	South America / Brazil / Sao Paulo / Lins
EPI_ISL_1096135	2021 Feb 9	2021 Feb 28	South America / Brazil / Sao Paulo / Araraquara
EPI_ISL_1096136	2021 Feb 10	2021 Feb 28	South America / Brazil / Sao Paulo / Jau
EPI_ISL_1096262	2021 Feb 12	2021 Feb 28	South America / French Guiana / Cayenne
EPI_ISL_1121307	2021 Feb 13	2021 Mar 3	South America / Brazil / Sao Paulo / Cajamar
EPI_ISL_1121308	2021 Feb 11	2021 Mar 3	South America / Brazil / Sao Paulo / Sao Paulo
EPI_ISL_1121309	2021 Feb 10	2021 Mar 3	South America / Brazil / Sao Paulo / Osasco
EPI_ISL_1121310	2021 Feb 8	2021 Mar 3	South America / Brazil / Sao Paulo / Lins
EPI_ISL_1121311	2021 Feb 6	2021 Mar 3	South America / Brazil / Sao Paulo / Mococa
EPI_ISL_1121312	2021 Feb 5	2021 Mar 3	South America / Brazil / Sao Paulo / Lins
EPI_ISL_1121313	2021 Feb 5	2021 Mar 3	South America / Brazil / Sao Paulo / Lins
EPI_ISL_1121314	2021 Feb 5	2021 Mar 3	South America / Brazil / Sao Paulo / Lins
EPI_ISL_1121315	2021 Jan 16	2021 Mar 3	South America / Brazil / Sao Paulo / Borebi
EPI_ISL_1121316	2021 Jan 10	2021 Mar 3	South America / Brazil / Rio Grande do Sul / Porto Alegre
EPI_ISL_1123373	2021 Feb 15	2021 Mar 3	South America / Brazil / Sao Paulo / Biritiba Mirim
EPI_ISL_1139070	2021 Jan 20	2021 Mar 4	South America / Brazil / Sao Paulo / Araraquara
EPI_ISL_1164970	2021 Feb 5	2021 Mar 5	South America / Brazil / Ceara
EPI_ISL_1164971	2021 Feb 5	2021 Mar 5	South America / Brazil / Ceara
EPI_ISL_1164972	2021 Feb 2	2021 Mar 5	South America / Brazil / Para
EPI_ISL_1164973	2021 Jan 29	2021 Mar 5	South America / Brazil / Ceara
EPI_ISL_1164978	2021 Jan 19	2021 Mar 5	South America / Brazil / Para
EPI_ISL_1164980	2021 Jan 18	2021 Mar 5	South America / Brazil / Ceara
EPI_ISL_1164984	2021 Jan 16	2021 Mar 5	South America / Brazil / Amapa
EPI_ISL_1164991	2021 Jan 7	2021 Mar 5	South America / Brazil / Paraiba
EPI_ISL_1166615	2021 Jan 18	2021 Mar 5	South America / Brazil / Amazonas
EPI_ISL_1171619	2021 Feb 15	2021 Mar 7	South America / Brazil / Sao Paulo / Presidente Venceslau
EPI_ISL_1171648	2021 Jan 5	2021 Mar 7	South America / Brazil / Sao Paulo / Mococa
EPI_ISL_1171649	2021 Jan 5	2021 Mar 7	South America / Brazil / Sao Paulo / Mococa
EPI_ISL_1171650	2021 Jan 7	2021 Mar 7	South America / Brazil / Sao Paulo / Mococa
EPI_ISL_1171653	2021 Feb 5	2021 Mar 7	South America / Brazil / Sao Paulo / Presidente Prudente
EPI_ISL_1171656	2021 Feb 17	2021 Mar 7	South America / Brazil / Sao Paulo / Presidente Prudente
EPI_ISL_1171657	2021 Feb 17	2021 Mar 7	South America / Brazil / Sao Paulo / Presidente Prudente
EPI_ISL_1171658	2021 Feb 15	2021 Mar 7	South America / Brazil / Sao Paulo / Presidente Prudente
EPI_ISL_1171666	2021 Feb 16	2021 Mar 7	South America / Brazil / Sao Paulo / Martinopolis
EPI_ISL_1171672	2021 Jan 26	2021 Mar 7	South America / Brazil / Sao Paulo / Tarabai
EPI_ISL_1171674	2021 Feb 12	2021 Mar 7	South America / Brazil / Sao Paulo / Dracena
EPI_ISL_1182543	2021 Feb 8	2021 Mar 8	South America / Brazil / Minas Gerais / Coromandel
EPI_ISL_1182544	2021 Jan 30	2021 Mar 8	South America / Brazil / Minas Gerais / Belo Horizonte
EPI_ISL_1182545	2021 Jan 30	2021 Mar 8	South America / Brazil / Minas Gerais / Belo Horizonte
EPI_ISL_1196296	2021 Feb 5	2021 Mar 10	South America / Brazil / Sao Paulo / Mococa
EPI_ISL_1196299	2021 Feb 4	2021 Mar 10	South America / Brazil / Sao Paulo / Marilia
EPI_ISL_1201884	2021 Jan 21	2021 Mar 10	South America / Brazil / Sao Paulo / Guarulhos
EPI_ISL_1201893	2021 Feb 18	2021 Mar 10	South America / Brazil / Sao Paulo / Avare

Sample identification	Date collected	Date uploaded	Collection locations
EPI_ISL_1213168	2021 Feb 14	2021 Mar 11	South America / Brazil / Bahia / Ilheus
EPI_ISL_1213170	2021 Feb 8	2021 Mar 11	South America / Brazil / Bahia / Itabuna
EPI_ISL_1213171	2021 Feb 3	2021 Mar 11	South America / Brazil / Rio de Janeiro / Rio de janeiro
EPI_ISL_1213173	2021 Feb 1	2021 Mar 11	South America / Brazil / Rio Grande do Norte / Natal
EPI_ISL_1213175	2021 Feb 1	2021 Mar 11	South America / Brazil / Rio Grande do Norte / Natal
EPI_ISL_1213177	2021 Feb 1	2021 Mar 11	South America / Brazil / Rio Grande do Norte / Natal
EPI_ISL_1213178	2021 Feb 1	2021 Mar 11	South America / Brazil / Rio Grande do Norte / Natal
EPI_ISL_1213180	2021 Feb 1	2021 Mar 11	South America / Brazil / Rio Grande do Norte / Natal
EPI_ISL_1213182	2021 Feb 1	2021 Mar 11	South America / Brazil / Rio Grande do Norte / Natal
EPI_ISL_1213183	2021 Jan 29	2021 Mar 11	South America / Brazil / Rio Grande do Norte / Natal
EPI_ISL_1213185	2021 Jan 28	2021 Mar 11	South America / Brazil / Rio Grande do Norte / Natal
EPI_ISL_1213187	2021 Jan 28	2021 Mar 11	South America / Brazil / Rio Grande do Norte / Natal
EPI_ISL_1213189	2021 Jan 27	2021 Mar 11	South America / Brazil / Paraíba / Conde
EPI_ISL_1213190	2021 Jan 26	2021 Mar 11	South America / Brazil / Amazonas / Manaus
EPI_ISL_1213192	2021 Jan 26	2021 Mar 11	South America / Brazil / Rio Grande do Norte / Natal
EPI_ISL_1213194	2021 Jan 26	2021 Mar 11	South America / Brazil / Rio Grande do Norte / Natal
EPI_ISL_1213196	2021 Jan 26	2021 Mar 11	South America / Brazil / Rio Grande do Norte / Natal
EPI_ISL_1213197	2021 Jan 26	2021 Mar 11	South America / Brazil / Rio Grande do Norte / Natal
EPI_ISL_1213199	2021 Jan 26	2021 Mar 11	South America / Brazil / Rio Grande do Norte / Natal
EPI_ISL_1213201	2021 Jan 21	2021 Mar 11	South America / Brazil / Paraíba / Joao pessoa
EPI_ISL_1213202	2021 Jan 18	2021 Mar 11	South America / Brazil / Amazonas / Manaus
EPI_ISL_1213204	2021 Jan 18	2021 Mar 11	South America / Brazil / Amazonas / Manaus
EPI_ISL_1213206	2021 Jan 18	2021 Mar 11	South America / Brazil / Amazonas / Manaus
EPI_ISL_1213207	2021 Jan 11	2021 Mar 11	South America / Brazil / Paraíba / Inga
EPI_ISL_1219021	2021 Feb 24	2021 Mar 11	South America / Brazil / Sao Paulo / Sao Paulo
EPI_ISL_1219025	2021 Feb 2	2021 Mar 11	South America / Brazil / Sao Paulo / Guapiara
EPI_ISL_1219029	2021 Jan 23	2021 Mar 11	South America / Brazil / Sao Paulo / Guarulhos
EPI_ISL_1219030	2021 Jan 23	2021 Mar 11	South America / Brazil / Sao Paulo / Guarulhos
EPI_ISL_1219033	2021 Jan 22	2021 Mar 11	South America / Brazil / Sao Paulo / Guarulhos
EPI_ISL_1219036	2021 Jan 21	2021 Mar 11	South America / Brazil / Sao Paulo / Guarulhos
EPI_ISL_1219133	2021 Jan 26	2021 Mar 11	South America / Brazil / Parana / Curitiba
EPI_ISL_1219134	2021 Jan 27	2021 Mar 11	South America / Brazil / Alagoas / Maceio
EPI_ISL_1219135	2021 Jan 27	2021 Mar 11	South America / Brazil / Alagoas / Maceio
EPI_ISL_1219136	2021 Feb 15	2021 Mar 11	South America / Brazil / Bahia / Salvador
EPI_ISL_1239012	2021 Jan 26	2021 Mar 13	South America / Brazil / Amazonas / Manaus
EPI_ISL_1239111	2021 Feb 13	2021 Mar 14	South America / Brazil / Rio de Janeiro / Petropolis
EPI_ISL_1239119	2021 Jan 26	2021 Mar 14	South America / Brazil / Espirito Santo / Lorena
EPI_ISL_1239137	2021 Mar 1	2021 Mar 14	South America / Brazil / Minas Gerais / Monte Alegre de Minas
EPI_ISL_1239138	2021 Mar 2	2021 Mar 14	South America / Brazil / Minas Gerais / Montes Claros
EPI_ISL_1261683	2021 Jan 19	2021 Mar 16	South America / Brazil / Amazonas
EPI_ISL_1261684	2021 Feb 15	2021 Mar 16	South America / Brazil / Ceara
EPI_ISL_1261685	2021 Jan 20	2021 Mar 16	South America / Brazil / Amazonas
EPI_ISL_1261686	2021 Jan 31	2021 Mar 16	South America / Brazil / Amapa
EPI_ISL_1261687	2021 Feb 15	2021 Mar 16	South America / Brazil / Roraima
EPI_ISL_1261688	2021 Feb 15	2021 Mar 16	South America / Brazil / Amapa
EPI_ISL_1261689	2021 Feb 15	2021 Mar 16	South America / Brazil / Amapa
EPI_ISL_1261690	2021 Jan 11	2021 Mar 16	South America / Brazil / Amazonas
EPI_ISL_1261691	2021 Feb 11	2021 Mar 16	South America / Brazil / Para
EPI_ISL_1261692	2021 Feb 8	2021 Mar 16	South America / Brazil / Amapa
EPI_ISL_1261693	2021 Feb 15	2021 Mar 16	South America / Brazil / Ceara
EPI_ISL_1261694	2021 Jan 12	2021 Mar 16	South America / Brazil / Amazonas
EPI_ISL_1262913	2021 Feb 21	2021 Mar 16	South America / French Guiana / Cayenne
EPI_ISL_1289960	2021 Jan 11	2021 Mar 18	South America / Brazil / Sao Paulo / Ribeirao Preto
EPI_ISL_1293053	2021 Jan 18	2021 Mar 19	South America / Brazil / Rondonia / Porto Velho
EPI_ISL_1293054	2021 Jan 18	2021 Mar 19	South America / Brazil / Rondonia / Porto Velho
EPI_ISL_1293055	2021 Jan 18	2021 Mar 19	South America / Brazil / Rondonia / Porto Velho
EPI_ISL_1293059	2021 Feb 2	2021 Mar 19	South America / Brazil / Sao Paulo / Guapiara
EPI_ISL_1293063	2021 Feb 6	2021 Mar 19	South America / Brazil / Sao Paulo / Salto
EPI_ISL_1293067	2021 Feb 8	2021 Mar 19	South America / Brazil / Sao Paulo / Guapiara
EPI_ISL_1293075	2021 Feb 3	2021 Mar 19	South America / Brazil / Sao Paulo / Sorocaba
EPI_ISL_1293077	2021 Feb 3	2021 Mar 19	South America / Brazil / Sao Paulo / Tiete
EPI_ISL_1293080	2021 Feb 1	2021 Mar 19	South America / Brazil / Sao Paulo / Porto Feliz
EPI_ISL_1303502	2021 Jan 19	2021 Mar 21	South America / Brazil / Rondonia / Porto Velho
EPI_ISL_1303503	2021 Jan 18	2021 Mar 21	South America / Brazil / Rondonia / Porto Velho
EPI_ISL_1303506	2021 Jan 22	2021 Mar 21	South America / Brazil / Distrito Federal / Planaltina
EPI_ISL_1303509	2021 Feb 1	2021 Mar 21	South America / Brazil / Tocantins / Palmas
EPI_ISL_1303512	2021 Jan 25	2021 Mar 21	South America / Brazil / Goias / Goiania
EPI_ISL_1303513	2021 Jan 13	2021 Mar 21	South America / Brazil / Goias / Turvania

Sample identification	Date collected	Date uploaded	Collection locations
EPI_ISL_1303514	2021 Jan 19	2021 Mar 21	South America / Brazil / Goias / Mangueira
EPI_ISL_1303515	2021 Jan 13	2021 Mar 21	South America / Brazil / Goias / Britania
EPI_ISL_1303517	2021 Feb 5	2021 Mar 21	South America / Brazil / Goias / Jaragua
EPI_ISL_1303520	2021 Feb 10	2021 Mar 21	South America / Brazil / Sao Paulo / Meridiano
EPI_ISL_1303522	2021 Feb 12	2021 Mar 21	South America / Brazil / Sao Paulo / Votuporanga
EPI_ISL_1303525	2021 Feb 16	2021 Mar 21	South America / Brazil / Sao Paulo / Ipigua
EPI_ISL_1303529	2021 Feb 12	2021 Mar 21	South America / Brazil / Sao Paulo / Sao Jose do Rio Preto
EPI_ISL_1303536	2021 Feb 15	2021 Mar 21	South America / Brazil / Sao Paulo / Sao Paulo
EPI_ISL_1303537	2021 Feb 15	2021 Mar 21	South America / Brazil / Sao Paulo / Sao Paulo
EPI_ISL_1303539	2021 Feb 15	2021 Mar 21	South America / Brazil / Sao Paulo / Sao Paulo
EPI_ISL_1303541	2021 Feb 15	2021 Mar 21	South America / Brazil / Sao Paulo / Sao Paulo
EPI_ISL_1303542	2021 Feb 15	2021 Mar 21	South America / Brazil / Sao Paulo / Ceario Lange
EPI_ISL_1303543	2021 Feb 15	2021 Mar 21	South America / Brazil / Sao Paulo / Sao Paulo
EPI_ISL_1303545	2021 Feb 15	2021 Mar 21	South America / Brazil / Sao Paulo / Maua
EPI_ISL_1303546	2021 Feb 15	2021 Mar 21	South America / Brazil / Sao Paulo / Sao Paulo
EPI_ISL_1303547	2021 Feb 15	2021 Mar 21	South America / Brazil / Sao Paulo / Diadema
EPI_ISL_1303548	2021 Feb 15	2021 Mar 21	South America / Brazil / Sao Paulo / Sao Paulo
EPI_ISL_1303549	2021 Feb 16	2021 Mar 21	South America / Brazil / Sao Paulo / Sao Paulo
EPI_ISL_1321468	2021 Jan 28	2021 Mar 23	South America / Chile / Maule / San Clemente
EPI_ISL_1321469	2021 Feb 25	2021 Mar 23	South America / Chile / Maule / Talca
EPI_ISL_1321470	2021 Feb 24	2021 Mar 23	South America / Chile / Maule / Molina
EPI_ISL_1321471	2021 Feb 28	2021 Mar 23	South America / Chile / Valparaiso / Valparaiso
EPI_ISL_1321472	2021 Mar 10	2021 Mar 23	South America / Chile / Region Metropolitana de Santiago / La Pintana
EPI_ISL_1321473	2021 Mar 11	2021 Mar 23	South America / Chile / Region Metropolitana de Santiago / La Granja
EPI_ISL_1358285	2021 Feb 24	2021 Mar 25	South America / Brazil / Sao Paulo / Sao Paulo
EPI_ISL_1358286	2021 Feb 24	2021 Mar 25	South America / Brazil / Sao Paulo / Sao Paulo
EPI_ISL_1358287	2021 Mar 7	2021 Mar 25	South America / Brazil / Sao Paulo / Sao Paulo
EPI_ISL_1358288	2021 Mar 4	2021 Mar 25	South America / Brazil / Sao Paulo / Aracatuba
EPI_ISL_1358289	2021 Mar 4	2021 Mar 25	South America / Brazil / Sao Paulo / Aracatuba
EPI_ISL_1358290	2021 Mar 4	2021 Mar 25	South America / Brazil / Sao Paulo / Aracatuba
EPI_ISL_1358292	2021 Feb 12	2021 Mar 25	South America / Brazil / Sao Paulo / Diadema
EPI_ISL_1358293	2021 Feb 11	2021 Mar 25	South America / Brazil / Sao Paulo / Diadema
EPI_ISL_1358300	2021 Feb 22	2021 Mar 25	South America / Brazil / Tocantins / Gurupi
EPI_ISL_1358301	2021 Feb 22	2021 Mar 25	South America / Brazil / Tocantins / Palmas
EPI_ISL_1358304	2021 Feb 10	2021 Mar 25	South America / Brazil / Mato Grosso do Sul / Campo Grande
EPI_ISL_1358318	2021 Feb 27	2021 Mar 25	South America / Brazil / Sao Paulo / Sao Paulo
EPI_ISL_1358319	2021 Feb 27	2021 Mar 25	South America / Brazil / Sao Paulo / Sao Paulo
EPI_ISL_1358320	2021 Feb 26	2021 Mar 25	South America / Brazil / Sao Paulo / Sao Paulo
EPI_ISL_1358321	2021 Feb 16	2021 Mar 25	South America / Brazil / Sao Paulo / Diadema
EPI_ISL_1365747	2021 Mar 12	2021 Mar 26	South America / Brazil / Sao Paulo / Sao Paulo
EPI_ISL_1381045	2021 Feb 11	2021 Mar 28	South America / Brazil / Sao Paulo / Diadema
EPI_ISL_1381047	2021 Feb 10	2021 Mar 28	South America / Brazil / Sao Paulo / Diadema
EPI_ISL_1381048	2021 Feb 10	2021 Mar 28	South America / Brazil / Sao Paulo / Diadema
EPI_ISL_1381050	2021 Feb 11	2021 Mar 28	South America / Brazil / Sao Paulo / Maua
EPI_ISL_1381052	2021 Feb 11	2021 Mar 28	South America / Brazil / Sao Paulo / Maua
EPI_ISL_1381055	2021 Feb 11	2021 Mar 28	South America / Brazil / Sao Paulo / Maua
EPI_ISL_1381056	2021 Feb 11	2021 Mar 28	South America / Brazil / Sao Paulo / Maua
EPI_ISL_1381057	2021 Feb 11	2021 Mar 28	South America / Brazil / Sao Paulo / Rio Grande da Serra
EPI_ISL_1381059	2021 Feb 11	2021 Mar 28	South America / Brazil / Sao Paulo / Santo Andre
EPI_ISL_1381060	2021 Feb 10	2021 Mar 28	South America / Brazil / Sao Paulo / Sao Bernardo do Campo
EPI_ISL_1381061	2021 Feb 10	2021 Mar 28	South America / Brazil / Sao Paulo / Sao Bernardo do Campo
EPI_ISL_1381062	2021 Feb 10	2021 Mar 28	South America / Brazil / Sao Paulo / Sao Bernardo do Campo
EPI_ISL_1381063	2021 Feb 10	2021 Mar 28	South America / Brazil / Sao Paulo / Sao Bernardo do Campo
EPI_ISL_1381065	2021 Feb 10	2021 Mar 28	South America / Brazil / Sao Paulo / Sao Bernardo do Campo
EPI_ISL_1381068	2021 Mar 2	2021 Mar 28	South America / Brazil / Sao Paulo / Sao Paulo
EPI_ISL_1381069	2021 Mar 2	2021 Mar 28	South America / Brazil / Sao Paulo / Sao Paulo
EPI_ISL_1381070	2021 Mar 12	2021 Mar 28	South America / Brazil / Sao Paulo / Sao Paulo
EPI_ISL_1381071	2021 Mar 12	2021 Mar 28	South America / Brazil / Sao Paulo / Sao Paulo
EPI_ISL_1381214	2021 Feb 26	2021 Mar 28	South America / French Guiana / Cayenne
EPI_ISL_1381216	2021 Mar 1	2021 Mar 28	South America / French Guiana / Cayenne
EPI_ISL_1381217	2021 Mar 3	2021 Mar 28	South America / French Guiana / Cayenne
EPI_ISL_1381222	2021 Mar 1	2021 Mar 28	South America / French Guiana / Cayenne
EPI_ISL_1402431	2021 Jan 7	2021 Mar 30	South America / Brazil / Amazonas / Manaus
EPI_ISL_1443196	2020 Dec 28	2021 Apr 1	South America / Brazil / Bahia / Salvador
EPI_ISL_1443197	2020 Dec 28	2021 Apr 1	South America / Brazil / Bahia / Salvador
EPI_ISL_1443198	2020 Dec 28	2021 Apr 1	South America / Brazil / Bahia / Salvador
EPI_ISL_1464627	2021 Mar 9	2021 Apr 2	South America / Brazil / Sao Paulo / Sao Paulo
EPI_ISL_1464628	2021 Mar 11	2021 Apr 2	South America / Brazil / Sao Paulo / Sao Paulo

Sample identification	Date collected	Date uploaded	Collection locations
EPI_ISL_1464630	2021 Mar 2	2021 Apr 2	South America / Brazil / Sao Paulo / Sao Paulo
EPI_ISL_1464631	2021 Mar 2	2021 Apr 2	South America / Brazil / Sao Paulo / Sao Paulo
EPI_ISL_1464632	2021 Mar 15	2021 Apr 2	South America / Brazil / Sao Paulo / Sao Paulo
EPI_ISL_1464633	2021 Mar 15	2021 Apr 2	South America / Brazil / Sao Paulo / Sao Paulo
EPI_ISL_1464634	2021 Mar 14	2021 Apr 2	South America / Brazil / Sao Paulo / Sao Paulo
EPI_ISL_1464635	2021 Mar 13	2021 Apr 2	South America / Brazil / Sao Paulo / Sao Paulo
EPI_ISL_1464636	2021 Mar 12	2021 Apr 2	South America / Brazil / Sao Paulo / Sao Paulo
EPI_ISL_1464637	2021 Mar 12	2021 Apr 2	South America / Brazil / Sao Paulo / Sao Paulo
EPI_ISL_1464638	2021 Mar 12	2021 Apr 2	South America / Brazil / Sao Paulo / Sao Paulo
EPI_ISL_1464639	2021 Mar 11	2021 Apr 2	South America / Brazil / Sao Paulo / Sao Paulo
EPI_ISL_1464640	2021 Mar 11	2021 Apr 2	South America / Brazil / Sao Paulo / Sao Paulo
EPI_ISL_1464641	2021 Mar 11	2021 Apr 2	South America / Brazil / Sao Paulo / Sao Paulo
EPI_ISL_1464642	2021 Mar 11	2021 Apr 2	South America / Brazil / Sao Paulo / Sao Paulo
EPI_ISL_1464643	2021 Mar 11	2021 Apr 2	South America / Brazil / Sao Paulo / Sao Paulo
EPI_ISL_1464644	2021 Mar 11	2021 Apr 2	South America / Brazil / Sao Paulo / Sao Paulo
EPI_ISL_1464645	2021 Mar 11	2021 Apr 2	South America / Brazil / Sao Paulo / Sao Paulo
EPI_ISL_1464646	2021 Mar 10	2021 Apr 2	South America / Brazil / Sao Paulo / Sao Paulo
EPI_ISL_1464647	2021 Mar 10	2021 Apr 2	South America / Brazil / Sao Paulo / Sao Paulo
EPI_ISL_1464648	2021 Mar 10	2021 Apr 2	South America / Brazil / Sao Paulo / Sao Paulo
EPI_ISL_1464649	2021 Mar 10	2021 Apr 2	South America / Brazil / Sao Paulo / Sao Paulo
EPI_ISL_1464650	2021 Mar 9	2021 Apr 2	South America / Brazil / Sao Paulo / Sao Paulo
EPI_ISL_1464651	2021 Mar 9	2021 Apr 2	South America / Brazil / Sao Paulo / Sao Paulo
EPI_ISL_1464652	2021 Mar 8	2021 Apr 2	South America / Brazil / Sao Paulo / Sao Paulo
EPI_ISL_1464653	2021 Mar 7	2021 Apr 2	South America / Brazil / Sao Paulo / Sao Paulo
EPI_ISL_1464654	2021 Mar 6	2021 Apr 2	South America / Brazil / Sao Paulo / Sao Paulo
EPI_ISL_1464655	2021 Mar 6	2021 Apr 2	South America / Brazil / Sao Paulo / Sao Paulo
EPI_ISL_1464656	2021 Mar 6	2021 Apr 2	South America / Brazil / Sao Paulo / Sao Paulo
EPI_ISL_1464657	2021 Mar 6	2021 Apr 2	South America / Brazil / Sao Paulo / Sao Paulo
EPI_ISL_1464658	2021 Mar 5	2021 Apr 2	South America / Brazil / Sao Paulo / Sao Paulo
EPI_ISL_1464659	2021 Mar 5	2021 Apr 2	South America / Brazil / Sao Paulo / Sao Paulo
EPI_ISL_1464660	2021 Mar 4	2021 Apr 2	South America / Brazil / Sao Paulo / Sao Paulo
EPI_ISL_1464661	2021 Mar 4	2021 Apr 2	South America / Brazil / Sao Paulo / Sao Paulo
EPI_ISL_1464662	2021 Mar 4	2021 Apr 2	South America / Brazil / Sao Paulo / Sao Paulo
EPI_ISL_1464663	2021 Mar 3	2021 Apr 2	South America / Brazil / Sao Paulo / Sao Paulo
EPI_ISL_1464664	2021 Mar 3	2021 Apr 2	South America / Brazil / Sao Paulo / Sao Paulo
EPI_ISL_1464665	2021 Mar 2	2021 Apr 2	South America / Brazil / Sao Paulo / Sao Paulo
EPI_ISL_1464666	2021 Mar 2	2021 Apr 2	South America / Brazil / Sao Paulo / Sao Paulo
EPI_ISL_1464667	2021 Mar 2	2021 Apr 2	South America / Brazil / Sao Paulo / Sao Paulo
EPI_ISL_1464668	2021 Mar 2	2021 Apr 2	South America / Brazil / Sao Paulo / Sao Paulo
EPI_ISL_1464669	2021 Mar 2	2021 Apr 2	South America / Brazil / Sao Paulo / Sao Paulo
EPI_ISL_1464670	2021 Mar 2	2021 Apr 2	South America / Brazil / Sao Paulo / Sao Paulo
EPI_ISL_1464671	2021 Mar 2	2021 Apr 2	South America / Brazil / Sao Paulo / Sao Paulo
EPI_ISL_1465188	2021 Feb 24	2021 Apr 2	South America / Brazil / Maranhao / Sao Luis
EPI_ISL_1465189	2021 Feb 23	2021 Apr 2	South America / Brazil / Maranhao / Sao Luis
EPI_ISL_1465191	2021 Feb 23	2021 Apr 2	South America / Brazil / Maranhao / Sao Luis
EPI_ISL_1465192	2021 Feb 22	2021 Apr 2	South America / Brazil / Maranhao / Miranda do Norte
EPI_ISL_1465194	2021 Feb 20	2021 Apr 2	South America / Brazil / Maranhao / Santa Ines
EPI_ISL_1465195	2021 Feb 19	2021 Apr 2	South America / Brazil / Maranhao / Sao Jose de Ribamar
EPI_ISL_1465196	2021 Feb 19	2021 Apr 2	South America / Brazil / Maranhao / Sao Luis
EPI_ISL_1465198	2021 Feb 18	2021 Apr 2	South America / Brazil / Maranhao / Timon
EPI_ISL_1465199	2021 Feb 12	2021 Apr 2	South America / Brazil / Maranhao / Sao Jose de Ribamar
EPI_ISL_1465201	2021 Feb 12	2021 Apr 2	South America / Brazil / Maranhao / Sao Luis
EPI_ISL_1465202	2021 Feb 11	2021 Apr 2	South America / Brazil / Maranhao / Sao Luis
EPI_ISL_1465203	2021 Feb 9	2021 Apr 2	South America / Brazil / Maranhao / Sao Luis
EPI_ISL_1465205	2021 Feb 8	2021 Apr 2	South America / Brazil / Maranhao / Sao Luis
EPI_ISL_1465206	2021 Feb 7	2021 Apr 2	South America / Brazil / Maranhao / Imperatriz
EPI_ISL_1465208	2021 Feb 7	2021 Apr 2	South America / Brazil / Maranhao / Sao Luis
EPI_ISL_1465209	2021 Feb 7	2021 Apr 2	South America / Brazil / Maranhao / Sao Luis
EPI_ISL_1465210	2021 Feb 6	2021 Apr 2	South America / Brazil / Maranhao / Sao Luis
EPI_ISL_1465212	2021 Feb 5	2021 Apr 2	South America / Brazil / Maranhao / Sao Luis
EPI_ISL_1465213	2021 Feb 4	2021 Apr 2	South America / Brazil / Maranhao / Sao Jose de Ribamar
EPI_ISL_1465215	2021 Feb 3	2021 Apr 2	South America / Brazil / Maranhao / Sao Luis
EPI_ISL_1465216	2021 Feb 1	2021 Apr 2	South America / Brazil / Maranhao / Sao Luis
EPI_ISL_1465217	2021 Jan 30	2021 Apr 2	South America / Brazil / Maranhao / Sao Luis
EPI_ISL_1465219	2021 Jan 23	2021 Apr 2	South America / Brazil / Maranhao / Sao Luis
EPI_ISL_1465220	2021 Jan 23	2021 Apr 2	South America / Brazil / Parana / Maringa
EPI_ISL_1465221	2021 Jan 19	2021 Apr 2	South America / Brazil / Maranhao / Sao Luis
EPI_ISL_1465222	2021 Jan 18	2021 Apr 2	South America / Brazil / Maranhao / Caxias

Sample identification	Date collected	Date uploaded	Collection locations
EPI_ISL_1468413	2021 Mar 16	2021 Apr 3	South America / Brazil / Goias / Cidade Ocidental
EPI_ISL_1468414	2021 Mar 16	2021 Apr 3	South America / Brazil / Goias / Goiania
EPI_ISL_1468415	2021 Mar 16	2021 Apr 3	South America / Brazil / Goias / Goiania
EPI_ISL_1468416	2021 Feb 22	2021 Apr 3	South America / Brazil / Sao Paulo / Andradina
EPI_ISL_1468417	2021 Feb 2	2021 Apr 3	South America / Brazil / Sao Paulo / Aracatuba
EPI_ISL_1468418	2021 Jan 11	2021 Apr 3	South America / Brazil / Sao Paulo / Birigui
EPI_ISL_1468419	2021 Feb 24	2021 Apr 3	South America / Brazil / Sao Paulo / Aracatuba
EPI_ISL_1468420	2021 Feb 22	2021 Apr 3	South America / Brazil / Sao Paulo / Birigui
EPI_ISL_1468421	2021 Feb 22	2021 Apr 3	South America / Brazil / Sao Paulo / Birigui
EPI_ISL_1468422	2021 Feb 17	2021 Apr 3	South America / Brazil / Sao Paulo / Avanhandava
EPI_ISL_1468423	2021 Feb 17	2021 Apr 3	South America / Brazil / Sao Paulo / Avanhandava
EPI_ISL_1468424	2021 Feb 22	2021 Apr 3	South America / Brazil / Sao Paulo / Aracatuba
EPI_ISL_1468425	2021 Feb 16	2021 Apr 3	South America / Brazil / Sao Paulo / Birigui
EPI_ISL_1468426	2021 Feb 18	2021 Apr 3	South America / Brazil / Sao Paulo / Matao
EPI_ISL_1468427	2021 Feb 9	2021 Apr 3	South America / Brazil / Sao Paulo / Matao
EPI_ISL_1468428	2021 Jan 31	2021 Apr 3	South America / Brazil / Sao Paulo / Taguaritinga
EPI_ISL_1468429	2021 Feb 23	2021 Apr 3	South America / Brazil / Sao Paulo / Matao
EPI_ISL_1468430	2021 Feb 3	2021 Apr 3	South America / Brazil / Sao Paulo / Matao
EPI_ISL_1470421	2021 Mar 8	2021 Apr 5	South America / Chile / Ñuble / Chillan
EPI_ISL_1470429	2021 Mar 15	2021 Apr 5	South America / Chile / Tarapaca / Iquique
EPI_ISL_1470430	2021 Mar 15	2021 Apr 5	South America / Chile / Tarapaca / Iquique
EPI_ISL_1470436	2021 Mar 17	2021 Apr 5	South America / Chile / Region Metropolitana de Santiago / Melipilla
EPI_ISL_1470437	2021 Mar 17	2021 Apr 5	South America / Chile / Region Metropolitana de Santiago / Talagante
EPI_ISL_1470439	2021 Mar 17	2021 Apr 5	South America / Chile / Region Metropolitana de Santiago / Talagante
EPI_ISL_1470440	2021 Mar 17	2021 Apr 5	South America / Chile / Region Metropolitana de Santiago / Melipilla
EPI_ISL_1470442	2021 Mar 17	2021 Apr 5	South America / Chile / Region Metropolitana de Santiago / Melipilla
EPI_ISL_1470451	2021 Mar 22	2021 Apr 5	South America / Chile / Region Metropolitana de Santiago / San Bernardo
EPI_ISL_1470452	2021 Mar 22	2021 Apr 5	South America / Chile / Region Metropolitana de Santiago / San Bernardo
EPI_ISL_1470454	2021 Mar 22	2021 Apr 5	South America / Chile / Region Metropolitana de Santiago / Las Condes
EPI_ISL_1470457	2021 Mar 21	2021 Apr 5	South America / Chile / Region Metropolitana de Santiago / Huechuraba
EPI_ISL_1470463	2021 Mar 24	2021 Apr 5	South America / Chile / Maule / Talca
EPI_ISL_1470465	2021 Mar 24	2021 Apr 5	South America / Chile / Maule / Talca
EPI_ISL_1470477	2021 Mar 23	2021 Apr 5	South America / Chile / Los Lagos / Puerto Montt
EPI_ISL_1470478	2021 Mar 23	2021 Apr 5	South America / Chile / Los Lagos / Puerto Montt
EPI_ISL_1470491	2021 Mar 12	2021 Apr 5	South America / Chile / Region Metropolitana de Santiago / Puente Alto
EPI_ISL_1470497	2021 Mar 15	2021 Apr 5	South America / Chile / Region Metropolitana de Santiago / San Ramon
EPI_ISL_1470501	2021 Mar 16	2021 Apr 5	South America / Chile / Region Metropolitana de Santiago / Puente Alto
EPI_ISL_1470506	2021 Mar 17	2021 Apr 5	South America / Chile / Region Metropolitana de Santiago / San Ramon
EPI_ISL_1470508	2021 Mar 18	2021 Apr 5	South America / Chile / Region Metropolitana de Santiago / Puente Alto
EPI_ISL_1470517	2021 Mar 22	2021 Apr 5	South America / Chile / Region Metropolitana de Santiago / San Ramon
EPI_ISL_1470522	2021 Mar 24	2021 Apr 5	South America / Chile / Region Metropolitana de Santiago / La Pintana
EPI_ISL_1470523	2021 Mar 24	2021 Apr 5	South America / Chile / Region Metropolitana de Santiago / La Pintana
EPI_ISL_1470531	2021 Mar 28	2021 Apr 5	South America / Chile / Maule / Molina
EPI_ISL_1470534	2021 Mar 28	2021 Apr 5	South America / Chile / Maule / Molina
EPI_ISL_1470542	2021 Mar 26	2021 Apr 5	South America / Chile / Los Lagos / Puerto Montt
EPI_ISL_1470548	2021 Mar 30	2021 Apr 5	South America / Chile / Tarapaca / Iquique
EPI_ISL_1470549	2021 Mar 28	2021 Apr 5	South America / Chile / Tarapaca / Iquique
EPI_ISL_1470550	2021 Mar 27	2021 Apr 5	South America / Chile / Tarapaca / Iquique
EPI_ISL_1470551	2021 Mar 28	2021 Apr 5	South America / Chile / Tarapaca / Iquique
EPI_ISL_1470552	2021 Mar 28	2021 Apr 5	South America / Chile / Tarapaca / Iquique
EPI_ISL_1470554	2021 Mar 29	2021 Apr 5	South America / Chile / Tarapaca / Iquique
EPI_ISL_1470558	2021 Mar 24	2021 Apr 5	South America / Chile / Region Metropolitana de Santiago / Pudahuel
EPI_ISL_1493573	2021 Mar 13	2021 Apr 6	South America / Brazil / Goias / Rio Verde
EPI_ISL_1493574	2021 Mar 13	2021 Apr 6	South America / Brazil / Goias / Goiania
EPI_ISL_1493575	2021 Feb 28	2021 Apr 6	South America / Brazil / Goias / Simao
EPI_ISL_1493576	2021 Feb 27	2021 Apr 6	South America / Brazil / Goias / Goiania
EPI_ISL_1493577	2021 Feb 27	2021 Apr 6	South America / Brazil / Goias / Trindade
EPI_ISL_1493578	2021 Feb 26	2021 Apr 6	South America / Brazil / Rondonia / Porto Velho
EPI_ISL_1493579	2021 Feb 25	2021 Apr 6	South America / Brazil / Rondonia / Porto Velho
EPI_ISL_1493580	2021 Feb 9	2021 Apr 6	South America / Brazil / Sao Paulo / Orlandia
EPI_ISL_1493581	2021 Feb 7	2021 Apr 6	South America / Brazil / Sao Paulo / Taguaritinga
EPI_ISL_1493582	2021 Jan 31	2021 Apr 6	South America / Brazil / Sao Paulo / Orlandia
EPI_ISL_1494923	2021 Feb 23	2021 Apr 7	South America / Brazil / Sao Paulo / Ituverava
EPI_ISL_1494924	2021 Feb 9	2021 Apr 7	South America / Brazil / Rondonia / Porto Velho
EPI_ISL_1498380	2021 Feb 17	2021 Apr 7	South America / Brazil / Sao Paulo / Sao Paulo
EPI_ISL_1498916	2021 Feb 17	2021 Apr 7	South America / Brazil / Sao Paulo / Sao Paulo
EPI_ISL_1498917	2021 Feb 1	2021 Apr 7	South America / Brazil / Sao Paulo / Mirandopolis
EPI_ISL_1499105	2021 Feb 15	2021 Apr 7	South America / Brazil / Sao Paulo / Sao Paulo

Sample identification	Date collected	Date uploaded	Collection locations
EPI_ISL_1509639	2021 Feb 25	2021 Apr 8	South America / Brazil / Sao Paulo / Auriflama
EPI_ISL_1509720	2021 Feb 16	2021 Apr 8	South America / Brazil / Sao Paulo / Auriflama
EPI_ISL_1511643	2021 Jan 29	2021 Apr 8	South America / Brazil / Sao Paulo
EPI_ISL_1520108	2021 Mar 2	2021 Apr 9	South America / Brazil / Rondonia / Porto Velho
EPI_ISL_1520109	2021 Mar 3	2021 Apr 9	South America / Brazil / Rondonia / Porto Velho
EPI_ISL_1520123	2021 Feb 19	2021 Apr 9	South America / Brazil / Sao Paulo / Espirito Santo do Pinhal
EPI_ISL_1520129	2021 Feb 19	2021 Apr 9	South America / Brazil / Sao Paulo / Mococa
EPI_ISL_1520130	2021 Feb 19	2021 Apr 9	South America / Brazil / Sao Paulo / Mococa
EPI_ISL_1520131	2021 Feb 19	2021 Apr 9	South America / Brazil / Sao Paulo / Mococa
EPI_ISL_1520136	2021 Feb 19	2021 Apr 9	South America / Brazil / Sao Paulo / Mococa
EPI_ISL_1521321	2021 Feb 23	2021 Apr 9	South America / Suriname / Suriname
EPI_ISL_1521322	2021 Mar 22	2021 Apr 9	South America / Suriname / Suriname
EPI_ISL_1521323	2021 Mar 20	2021 Apr 9	South America / Suriname / Suriname
EPI_ISL_1521324	2021 Mar 1	2021 Apr 9	South America / Suriname / Suriname
EPI_ISL_1521325	2021 Mar 5	2021 Apr 9	South America / Suriname / Suriname
EPI_ISL_1521326	2021 Feb 22	2021 Apr 9	South America / Suriname / Suriname
EPI_ISL_1521327	2021 Mar 23	2021 Apr 9	South America / Suriname / Suriname
EPI_ISL_1521328	2021 Mar 23	2021 Apr 9	South America / Suriname / Suriname
EPI_ISL_1533609	2021 Feb 9	2021 Apr 10	South America / Brazil / Amazonas / Manaus
EPI_ISL_1533689	2021 Feb 19	2021 Apr 10	South America / Brazil / Sao Paulo / Mococa
EPI_ISL_1533690	2021 Feb 19	2021 Apr 10	South America / Brazil / Sao Paulo / Mococa
EPI_ISL_1533695	2021 Feb 11	2021 Apr 10	South America / Brazil / Sao Paulo / Campinas
EPI_ISL_1533696	2021 Jan 28	2021 Apr 10	South America / Brazil / Sao Paulo / Sao Paulo
EPI_ISL_1533699	2021 Feb 10	2021 Apr 10	South America / Brazil / Sao Paulo / Biritiba-Mirim
EPI_ISL_1533701	2021 Feb 15	2021 Apr 10	South America / Brazil / Sao Paulo / Sao Paulo
EPI_ISL_1533702	2021 Feb 16	2021 Apr 10	South America / Brazil / Sao Paulo / Sao Paulo
EPI_ISL_1533703	2021 Feb 20	2021 Apr 10	South America / Brazil / Sao Paulo / Campinas
EPI_ISL_1533704	2021 Feb 20	2021 Apr 10	South America / Brazil / Sao Paulo / Atibaia
EPI_ISL_1533706	2021 Feb 23	2021 Apr 10	South America / Brazil / Sao Paulo / Sao Paulo
EPI_ISL_1533707	2021 Feb 21	2021 Apr 10	South America / Brazil / Sao Paulo / Sao Paulo
EPI_ISL_1533709	2021 Mar 2	2021 Apr 10	South America / Brazil / Sao Paulo / Sao Paulo
EPI_ISL_1533710	2021 Mar 2	2021 Apr 10	South America / Brazil / Sao Paulo / Sao Paulo
EPI_ISL_1533711	2021 Feb 24	2021 Apr 10	South America / Brazil / Sao Paulo / Sao Paulo
EPI_ISL_1533712	2021 Mar 9	2021 Apr 10	South America / Brazil / Sao Paulo / Sao Paulo
EPI_ISL_1533713	2021 Mar 8	2021 Apr 10	South America / Brazil / Sao Paulo / Hortolandia
EPI_ISL_1533714	2021 Mar 6	2021 Apr 10	South America / Brazil / Sao Paulo / Osasco
EPI_ISL_1533715	2021 Mar 11	2021 Apr 10	South America / Brazil / Sao Paulo / Aracatuba
EPI_ISL_1533716	2021 Mar 10	2021 Apr 10	South America / Brazil / Sao Paulo / Campo Limpo Paulista
EPI_ISL_1533717	2021 Mar 7	2021 Apr 10	South America / Brazil / Sao Paulo / Hortolandia
EPI_ISL_1533718	2021 Mar 7	2021 Apr 10	South America / Brazil / Sao Paulo / Guarulhos
EPI_ISL_1533719	2021 Mar 7	2021 Apr 10	South America / Brazil / Sao Paulo / Holambra
EPI_ISL_1533720	2021 Mar 6	2021 Apr 10	South America / Brazil / Sao Paulo / Penapolis
EPI_ISL_1533722	2021 Mar 9	2021 Apr 10	South America / Brazil / Sao Paulo / Piracaia
EPI_ISL_1533723	2021 Mar 8	2021 Apr 10	South America / Brazil / Sao Paulo / Itaquaquecetuba
EPI_ISL_1533725	2021 Mar 3	2021 Apr 10	South America / Brazil / Sao Paulo / Guarulhos
EPI_ISL_1533726	2021 Mar 5	2021 Apr 10	South America / Brazil / Sao Paulo / Sao Paulo
EPI_ISL_1533727	2021 Mar 13	2021 Apr 10	South America / Brazil / Sao Paulo / Sao Paulo
EPI_ISL_1533992	2021 Jan 27	2021 Apr 10	South America / Brazil / Santa Catarina / Florianopolis
EPI_ISL_1533993	2021 Jan 28	2021 Apr 10	South America / Brazil / Santa Catarina / Palhoca
EPI_ISL_1533994	2021 Jan 28	2021 Apr 10	South America / Brazil / Santa Catarina / Palhoca
EPI_ISL_1533996	2021 Feb 1	2021 Apr 10	South America / Brazil / Santa Catarina / Sao Jose
EPI_ISL_1533999	2021 Feb 18	2021 Apr 10	South America / Brazil / Parana / Londrina
EPI_ISL_1534000	2021 Feb 19	2021 Apr 10	South America / Brazil / Parana / Londrina
EPI_ISL_1534001	2021 Feb 19	2021 Apr 10	South America / Brazil / Parana / Londrina
EPI_ISL_1534002	2021 Feb 23	2021 Apr 10	South America / Brazil / Santa Catarina / Joinville
EPI_ISL_1534003	2021 Feb 23	2021 Apr 10	South America / Brazil / Santa Catarina / Lages
EPI_ISL_1534004	2021 Feb 23	2021 Apr 10	South America / Brazil / Sergipe / Barra dos Coqueiros
EPI_ISL_1534007	2021 Feb 26	2021 Apr 10	South America / Brazil / Santa Catarina / Balneario Arroio do Silva
EPI_ISL_1534008	2021 Feb 26	2021 Apr 10	South America / Brazil / Santa Catarina / Joinville
EPI_ISL_1534009	2021 Feb 26	2021 Apr 10	South America / Brazil / Santa Catarina / Joinville
EPI_ISL_1534010	2021 Feb 26	2021 Apr 10	South America / Brazil / Santa Catarina / Joinville
EPI_ISL_1534011	2021 Mar 11	2021 Apr 10	South America / Brazil / Rio de Janeiro / Niteroi
EPI_ISL_1534012	2021 Mar 13	2021 Apr 10	South America / Brazil / Rio de Janeiro / Paraiba do Sul
EPI_ISL_1534013	2021 Mar 15	2021 Apr 10	South America / Brazil / Rio Grande do Sul / Porto Xavier
EPI_ISL_1534014	2021 Mar 16	2021 Apr 10	South America / Brazil / Rio de Janeiro / Rio de Janeiro
EPI_ISL_1534015	2021 Mar 22	2021 Apr 10	South America / Brazil / Rio de Janeiro / Rio de Janeiro
EPI_ISL_1534016	2021 Mar 23	2021 Apr 10	South America / Brazil / Rio de Janeiro / Rio de Janeiro
EPI_ISL_1534544	2021 Mar 24	2021 Apr 11	South America / Chile / Region Metropolitana de Santiago / La Granja

Sample identification	Date collected	Date uploaded	Collection locations
EPI_ISL_1534546	2021 Mar 18	2021 Apr 11	South America / Chile / Region Metropolitana de Santiago / Puente Alto
EPI_ISL_1534547	2021 Mar 17	2021 Apr 11	South America / Chile / Region Metropolitana de Santiago / Puente Alto
EPI_ISL_1534555	2021 Mar 17	2021 Apr 11	South America / Chile / Region Metropolitana de Santiago / San Jose De Maipo
EPI_ISL_1534556	2021 Mar 17	2021 Apr 11	South America / Chile / Region Metropolitana de Santiago / San Jose De Maipo
EPI_ISL_1534563	2021 Mar 12	2021 Apr 11	South America / Chile / Region Metropolitana de Santiago / San Jose De Maipo
EPI_ISL_1534567	2021 Mar 26	2021 Apr 11	South America / Chile / Region Metropolitana de Santiago / La Granja
EPI_ISL_1534570	2021 Mar 27	2021 Apr 11	South America / Chile / Region Metropolitana de Santiago / La Pintana
EPI_ISL_1534572	2021 Mar 27	2021 Apr 11	South America / Chile / Region Metropolitana de Santiago / San Ramon
EPI_ISL_1534575	2021 Mar 27	2021 Apr 11	South America / Chile / Region Metropolitana de Santiago / San Ramon
EPI_ISL_1534578	2021 Mar 27	2021 Apr 11	South America / Chile / Region Metropolitana de Santiago / Puente Alto
EPI_ISL_1534581	2021 Mar 28	2021 Apr 11	South America / Chile / Region Metropolitana de Santiago / La Granja
EPI_ISL_1534589	2021 Mar 20	2021 Apr 11	South America / Chile / Region Metropolitana de Santiago / La Granja
EPI_ISL_1534590	2021 Mar 24	2021 Apr 11	South America / Chile / Region Metropolitana de Santiago / La Pintana
EPI_ISL_1534593	2021 Mar 29	2021 Apr 11	South America / Chile / Region Metropolitana de Santiago / Santiago
EPI_ISL_1534596	2021 Mar 30	2021 Apr 11	South America / Chile / Region Metropolitana de Santiago / Pudahuel
EPI_ISL_1534601	2021 Mar 30	2021 Apr 11	South America / Chile / Region Metropolitana de Santiago / Santiago
EPI_ISL_1534602	2021 Mar 29	2021 Apr 11	South America / Chile / Tarapaca / Iquique
EPI_ISL_1534603	2021 Mar 30	2021 Apr 11	South America / Chile / Tarapaca / Iquique
EPI_ISL_1534604	2021 Mar 30	2021 Apr 11	South America / Chile / Tarapaca / Iquique
EPI_ISL_1534605	2021 Mar 28	2021 Apr 11	South America / Chile / Valparaiso / Valparaiso
EPI_ISL_1534606	2021 Mar 29	2021 Apr 11	South America / Chile / Valparaiso / Valparaiso
EPI_ISL_1540997	2021 Mar 14	2021 Apr 12	South America / Chile / Region Metropolitana de Santiago / San Jose De Maipo
EPI_ISL_1541006	2021 Mar 15	2021 Apr 12	South America / Chile / Region Metropolitana de Santiago / La Granja
EPI_ISL_1541007	2021 Mar 15	2021 Apr 12	South America / Chile / Region Metropolitana de Santiago / La Granja
EPI_ISL_1541015	2021 Mar 29	2021 Apr 12	South America / Chile / La Araucania / Temuco
EPI_ISL_1583672	2021 Mar 2	2021 Apr 14	South America / Brazil / Bahia
EPI_ISL_1583673	2021 Mar 3	2021 Apr 14	South America / Brazil / Bahia
EPI_ISL_1583674	2021 Feb 8	2021 Apr 14	South America / Brazil / Bahia
EPI_ISL_1583675	2021 Mar 2	2021 Apr 14	South America / Brazil / Bahia
EPI_ISL_1583676	2021 Mar 5	2021 Apr 14	South America / Brazil / Bahia
EPI_ISL_1583677	2021 Feb 6	2021 Apr 14	South America / Brazil / Bahia
EPI_ISL_1583679	2021 Mar 4	2021 Apr 14	South America / Brazil / Bahia
EPI_ISL_1583680	2021 Feb 19	2021 Apr 14	South America / Brazil / Bahia
EPI_ISL_1583681	2021 Mar 2	2021 Apr 14	South America / Brazil / Bahia
EPI_ISL_1583682	2021 Feb 8	2021 Apr 14	South America / Brazil / Bahia
EPI_ISL_1583683	2021 Feb 12	2021 Apr 14	South America / Brazil / Bahia
EPI_ISL_1583694	2021 Mar 12	2021 Apr 14	South America / Brazil / Bahia
EPI_ISL_1583697	2021 Feb 23	2021 Apr 14	South America / Brazil / Bahia
EPI_ISL_1583700	2021 Feb 12	2021 Apr 14	South America / Brazil / Bahia
EPI_ISL_1583719	2021 Feb 23	2021 Apr 14	South America / Brazil / Bahia
EPI_ISL_1583722	2021 Feb 23	2021 Apr 14	South America / Brazil / Bahia
EPI_ISL_1583725	2021 Mar 1	2021 Apr 14	South America / Brazil / Bahia
EPI_ISL_1583730	2021 Mar 5	2021 Apr 14	South America / Brazil / Bahia
EPI_ISL_1583733	2021 Feb 26	2021 Apr 14	South America / Brazil / Bahia
EPI_ISL_1591098	2021 Feb 22	2021 Apr 14	South America / Peru
EPI_ISL_1593463	2021 Mar 12	2021 Apr 15	South America / French Guiana / Cayenne
EPI_ISL_1593464	2021 Mar 15	2021 Apr 15	South America / French Guiana / Regina
EPI_ISL_1593466	2021 Mar 15	2021 Apr 15	South America / French Guiana / Grand Santi
EPI_ISL_1593470	2021 Mar 19	2021 Apr 15	South America / French Guiana / Cayenne
EPI_ISL_1593471	2021 Mar 19	2021 Apr 15	South America / French Guiana / Cayenne
EPI_ISL_1593980	2021 Mar 2	2021 Apr 15	South America / French Guiana / Regina
EPI_ISL_1593984	2021 Mar 5	2021 Apr 15	South America / French Guiana / Kourou
EPI_ISL_1608161	2021 Feb 7	2021 Apr 15	South America / Brazil / Bahia / Salvador
EPI_ISL_1608162	2021 Feb 6	2021 Apr 15	South America / Brazil / Bahia / Salvador
EPI_ISL_1608163	2021 Feb 10	2021 Apr 15	South America / Brazil / Bahia / Salvador
EPI_ISL_1608164	2021 Feb 16	2021 Apr 15	South America / Brazil / Bahia / Salvador
EPI_ISL_1608165	2021 Feb 8	2021 Apr 15	South America / Brazil / Bahia / Salvador
EPI_ISL_1608166	2021 Feb 17	2021 Apr 15	South America / Brazil / Bahia / Salvador
EPI_ISL_1608167	2021 Feb 12	2021 Apr 15	South America / Brazil / Bahia / Salvador
EPI_ISL_1608168	2021 Feb 13	2021 Apr 15	South America / Brazil / Bahia / Salvador
EPI_ISL_1608169	2021 Feb 20	2021 Apr 15	South America / Brazil / Bahia / Salvador
EPI_ISL_1608170	2021 Feb 23	2021 Apr 15	South America / Brazil / Bahia / Salvador
EPI_ISL_1608171	2021 Feb 21	2021 Apr 15	South America / Brazil / Bahia / Salvador
EPI_ISL_1625972	2021 Mar 12	2021 Apr 16	South America / Brazil / Sao Paulo / Sao Paulo

Sample identification	Date collected	Date uploaded	Collection locations
EPI_ISL_1625974	2021 Mar 15	2021 Apr 16	South America / Brazil / Sao Paulo / Aracatuba
EPI_ISL_1625975	2021 Mar 7	2021 Apr 16	South America / Brazil / Sao Paulo / Sumare
EPI_ISL_1625976	2021 Mar 8	2021 Apr 16	South America / Brazil / Sao Paulo / Divinolandia
EPI_ISL_1625978	2021 Feb 15	2021 Apr 16	South America / Brazil / Sao Paulo / Rio Claro
EPI_ISL_1625984	2021 Feb 18	2021 Apr 16	South America / Brazil / Sao Paulo / Araras
EPI_ISL_1625986	2021 Feb 18	2021 Apr 16	South America / Brazil / Sao Paulo / Rio Claro
EPI_ISL_1625987	2021 Feb 20	2021 Apr 16	South America / Brazil / Sao Paulo / Rio Claro
EPI_ISL_1625988	2021 Feb 20	2021 Apr 16	South America / Brazil / Sao Paulo / Rio Claro
EPI_ISL_1625989	2021 Feb 21	2021 Apr 16	South America / Brazil / Sao Paulo / Limeira
EPI_ISL_1625990	2021 Feb 21	2021 Apr 16	South America / Brazil / Sao Paulo / Leme
EPI_ISL_1625991	2021 Feb 23	2021 Apr 16	South America / Brazil / Sao Paulo / Aguas de Sao Pedro
EPI_ISL_1625992	2021 Feb 24	2021 Apr 16	South America / Brazil / Sao Paulo / Limeira
EPI_ISL_1625993	2021 Feb 26	2021 Apr 16	South America / Brazil / Sao Paulo / Leme
EPI_ISL_1625994	2021 Feb 26	2021 Apr 16	South America / Brazil / Sao Paulo / Leme
EPI_ISL_1625995	2021 Feb 26	2021 Apr 16	South America / Brazil / Sao Paulo / Rio Claro
EPI_ISL_1625997	2021 Feb 25	2021 Apr 16	South America / Brazil / Sao Paulo / Rio Claro
EPI_ISL_1625998	2021 Feb 25	2021 Apr 16	South America / Brazil / Sao Paulo / Rio Claro
EPI_ISL_1625999	2021 Feb 26	2021 Apr 16	South America / Brazil / Sao Paulo / Rio Claro
EPI_ISL_1626001	2021 Feb 28	2021 Apr 16	South America / Brazil / Sao Paulo / Rio Claro
EPI_ISL_1626002	2021 Feb 28	2021 Apr 16	South America / Brazil / Sao Paulo / Rio Claro
EPI_ISL_1626003	2021 Feb 28	2021 Apr 16	South America / Brazil / Sao Paulo / Rio Claro
EPI_ISL_1626004	2021 Feb 28	2021 Apr 16	South America / Brazil / Sao Paulo / Rio Claro
EPI_ISL_1626006	2021 Feb 27	2021 Apr 16	South America / Brazil / Sao Paulo / Rio Claro
EPI_ISL_1626606	2021 Feb 27	2021 Apr 16	South America / Brazil / Sao Paulo / Rio Claro
EPI_ISL_1626613	2021 Mar 26	2021 Apr 17	South America / Colombia / Bogota
EPI_ISL_1626615	2021 Mar 24	2021 Apr 17	South America / Colombia / Bogota
EPI_ISL_1626616	2021 Mar 29	2021 Apr 17	South America / Colombia / Bogota
EPI_ISL_1626618	2021 Mar 25	2021 Apr 17	South America / Colombia / Bogota
EPI_ISL_1628344	2021 Mar 14	2021 Apr 17	South America / Brazil / Sao Paulo / Sao Paulo
EPI_ISL_1628347	2021 Mar 13	2021 Apr 17	South America / Brazil / Sao Paulo / Registro
EPI_ISL_1628348	2021 Mar 12	2021 Apr 17	South America / Brazil / Sao Paulo / Registro
EPI_ISL_1628349	2021 Mar 13	2021 Apr 17	South America / Brazil / Sao Paulo / Registro
EPI_ISL_1628350	2021 Mar 13	2021 Apr 17	South America / Brazil / Sao Paulo / Jacupiranga
EPI_ISL_1628351	2021 Mar 13	2021 Apr 17	South America / Brazil / Sao Paulo / Registro
EPI_ISL_1628352	2021 Mar 13	2021 Apr 17	South America / Brazil / Sao Paulo / Registro
EPI_ISL_1628353	2021 Mar 13	2021 Apr 17	South America / Brazil / Sao Paulo / Registro
EPI_ISL_1628354	2021 Mar 14	2021 Apr 17	South America / Brazil / Sao Paulo / Jacupiranga
EPI_ISL_1628355	2021 Mar 17	2021 Apr 17	South America / Brazil / Sao Paulo / Cajati
EPI_ISL_1628356	2021 Mar 17	2021 Apr 17	South America / Brazil / Sao Paulo / Cajati
EPI_ISL_1628357	2021 Mar 17	2021 Apr 17	South America / Brazil / Sao Paulo / Cajati
EPI_ISL_1628358	2021 Mar 17	2021 Apr 17	South America / Brazil / Sao Paulo / Cajati
EPI_ISL_1628359	2021 Mar 15	2021 Apr 17	South America / Brazil / Sao Paulo / Sao Joao da Boa Vista
EPI_ISL_1628360	2021 Mar 17	2021 Apr 17	South America / Brazil / Sao Paulo / Pariquera Acu
EPI_ISL_1628361	2021 Mar 17	2021 Apr 17	South America / Brazil / Sao Paulo / Pedro de Toledo
EPI_ISL_1628362	2021 Mar 17	2021 Apr 17	South America / Brazil / Sao Paulo / Pariquera Acu
EPI_ISL_1628363	2021 Mar 11	2021 Apr 17	South America / Brazil / Goias / Aparecida de Goiania
EPI_ISL_1628364	2021 Mar 16	2021 Apr 17	South America / Brazil / Goias / Goiania
EPI_ISL_1628365	2021 Mar 17	2021 Apr 17	South America / Brazil / Goias / Goiania
EPI_ISL_1628366	2021 Mar 18	2021 Apr 17	South America / Brazil / Sao Paulo / Patrocinio Paulista
EPI_ISL_1628367	2021 Mar 18	2021 Apr 17	South America / Brazil / Sao Paulo / Guaira
EPI_ISL_1628368	2021 Mar 16	2021 Apr 17	South America / Brazil / Sao Paulo / Pradopolis
EPI_ISL_1628369	2021 Mar 10	2021 Apr 17	South America / Brazil / Sao Paulo / Robeirao Preto
EPI_ISL_1628370	2021 Mar 9	2021 Apr 17	South America / Brazil / Sao Paulo / Taguaritinga
EPI_ISL_1628372	2021 Mar 15	2021 Apr 17	South America / Brazil / Sao Paulo / Morro Agudo
EPI_ISL_1628373	2021 Mar 22	2021 Apr 17	South America / Brazil / Sao Paulo / Bebedouro
EPI_ISL_1628374	2021 Mar 5	2021 Apr 17	South America / Brazil / Sao Paulo / Cravinhos
EPI_ISL_1628375	2021 Mar 18	2021 Apr 17	South America / Brazil / Sao Paulo / Guariba
EPI_ISL_1628376	2021 Mar 19	2021 Apr 17	South America / Brazil / Sao Paulo / Guariba
EPI_ISL_1628377	2021 Mar 9	2021 Apr 17	South America / Brazil / Sao Paulo / Ibitinga
EPI_ISL_1628378	2021 Mar 21	2021 Apr 17	South America / Brazil / Sao Paulo / Jardinopolis
EPI_ISL_1628379	2021 Mar 8	2021 Apr 17	South America / Brazil / Sao Paulo / Robeirao Preto
EPI_ISL_1628380	2021 Mar 19	2021 Apr 17	South America / Brazil / Sao Paulo / Ibitinga
EPI_ISL_1633373	2021 Mar 31	2021 Apr 19	South America / Chile / Los Rios / Lago Ranco
EPI_ISL_1633473	2021 Mar 13	2021 Apr 19	South America / Chile / Region Metropolitana de Santiago / La Pintana
EPI_ISL_1633475	2021 Mar 21	2021 Apr 19	South America / Chile / Region Metropolitana de Santiago / San Jose De Maipo
EPI_ISL_1633478	2021 Mar 28	2021 Apr 19	South America / Chile / Maule / Curico
EPI_ISL_1633494	2021 Mar 30	2021 Apr 19	South America / Chile / Region Metropolitana de Santiago / La Granja

Sample identification	Date collected	Date uploaded	Collection locations
EPI_ISL_1633502	2021 Mar 30	2021 Apr 19	South America / Chile / Region Metropolitana de Santiago / La Pintana
EPI_ISL_811149	2020 Dec 30	2021 Jan 13	South America / Brazil / Amazonas / Manaus
EPI_ISL_833136	2020 Dec 29	2021 Jan 17	South America / Brazil / Amazonas / Manaus
EPI_ISL_833137	2020 Dec 4	2021 Jan 17	South America / Brazil / Amazonas / Manaus
EPI_ISL_833138	2020 Dec 21	2021 Jan 17	South America / Brazil / Amazonas / Manaus
EPI_ISL_833139	2020 Dec 22	2021 Jan 17	South America / Brazil / Amazonas / Manaus
EPI_ISL_833140	2020 Dec 23	2021 Jan 17	South America / Brazil / Amazonas / Manaus
EPI_ISL_833167	2020 Dec 16	2021 Jan 17	South America / Brazil / Amazonas
EPI_ISL_833169	2020 Dec 23	2021 Jan 17	South America / Brazil / Amazonas
EPI_ISL_833170	2020 Dec 23	2021 Jan 17	South America / Brazil / Amazonas
EPI_ISL_833171	2020 Dec 17	2021 Jan 17	South America / Brazil / Amazonas
EPI_ISL_833172	2020 Dec 17	2021 Jan 17	South America / Brazil / Amazonas
EPI_ISL_833173	2020 Dec 18	2021 Jan 17	South America / Brazil / Amazonas
EPI_ISL_833174	2020 Dec 22	2021 Jan 17	South America / Brazil / Amazonas
EPI_ISL_872191	2021 Jan 15	2021 Jan 26	South America / Brazil / Sao Paulo / Sao Paulo
EPI_ISL_872192	2021 Jan 18	2021 Jan 26	South America / Brazil / Sao Paulo / Sao Paulo
EPI_ISL_875688	2021 Jan 4	2021 Jan 26	South America / Brazil / Sao Paulo / Sao Paulo
EPI_ISL_875689	2021 Jan 15	2021 Jan 26	South America / Brazil / Sao Paulo / Sao Paulo
EPI_ISL_904120	2021 Jan 10	2021 Jan 30	South America / Brazil / Para
EPI_ISL_904121	2021 Jan 8	2021 Jan 30	South America / Brazil / Para
EPI_ISL_906068	2021 Jan 15	2021 Jan 31	South America / Brazil / Sao Paulo / Campinas
EPI_ISL_906069	2021 Jan 15	2021 Jan 31	South America / Brazil / Amazonas / Manaus
EPI_ISL_906071	2021 Jan 19	2021 Jan 31	South America / Brazil / Amazonas / Manaus
EPI_ISL_906075	2021 Jan 19	2021 Jan 31	South America / Brazil / Amazonas / Manaus
EPI_ISL_906076	2021 Jan 19	2021 Jan 31	South America / Brazil / Amazonas / Manaus
EPI_ISL_906077	2021 Jan 19	2021 Jan 31	South America / Brazil / Amazonas / Manaus
EPI_ISL_906080	2021 Jan 22	2021 Jan 31	South America / Brazil / Amazonas / Manaus
EPI_ISL_906081	2021 Jan 22	2021 Jan 31	South America / Brazil / Amazonas / Manaus
EPI_ISL_918499	2020 Dec 21	2021 Feb 3	South America / Brazil / Amazonas
EPI_ISL_918500	2020 Dec 28	2021 Feb 3	South America / Brazil / Amazonas
EPI_ISL_918501	2020 Dec 28	2021 Feb 3	South America / Brazil / Amazonas
EPI_ISL_918502	2020 Dec 29	2021 Feb 3	South America / Brazil / Amazonas
EPI_ISL_918503	2020 Dec 28	2021 Feb 3	South America / Brazil / Amazonas
EPI_ISL_918504	2020 Dec 21	2021 Feb 3	South America / Brazil / Amazonas
EPI_ISL_918505	2020 Dec 30	2021 Feb 3	South America / Brazil / Amazonas
EPI_ISL_918506	2020 Dec 30	2021 Feb 3	South America / Brazil / Amazonas
EPI_ISL_918507	2021 Jan 5	2021 Feb 3	South America / Brazil / Amazonas
EPI_ISL_918508	2021 Jan 5	2021 Feb 3	South America / Brazil / Amazonas
EPI_ISL_918509	2021 Jan 6	2021 Feb 3	South America / Brazil / Amazonas
EPI_ISL_918510	2021 Jan 7	2021 Feb 3	South America / Brazil / Amazonas
EPI_ISL_918511	2020 Dec 30	2021 Feb 3	South America / Brazil / Amazonas
EPI_ISL_940614	2021 Jan 19	2021 Feb 5	South America / Brazil / Amazonas / Manaus
EPI_ISL_940615	2021 Jan 19	2021 Feb 5	South America / Brazil / Amazonas / Manaus
EPI_ISL_940616	2021 Jan 19	2021 Feb 5	South America / Brazil / Amazonas / Manaus
EPI_ISL_940617	2021 Jan 19	2021 Feb 5	South America / Brazil / Amazonas / Manaus
EPI_ISL_940618	2021 Jan 19	2021 Feb 5	South America / Brazil / Amazonas / Manaus
EPI_ISL_940619	2021 Jan 15	2021 Feb 5	South America / Brazil / Sao Paulo / Sao Paulo
EPI_ISL_940620	2021 Jan 14	2021 Feb 5	South America / Brazil / Amazonas / Manaus
EPI_ISL_940621	2021 Jan 14	2021 Feb 5	South America / Brazil / Sao Paulo / Sao Paulo
EPI_ISL_940622	2021 Jan 15	2021 Feb 5	South America / Brazil / Sao Paulo / Sao Paulo
EPI_ISL_940623	2021 Jan 8	2021 Feb 5	South America / Brazil / Amazonas / Manaus
EPI_ISL_940624	2021 Jan 15	2021 Feb 5	South America / Brazil / Amazonas / Manaus
EPI_ISL_940625	2021 Jan 19	2021 Feb 5	South America / Brazil / Sao Paulo / Sao Paulo
EPI_ISL_940626	2021 Jan 21	2021 Feb 5	South America / Brazil / Amazonas / Manaus
EPI_ISL_940627	2021 Jan 22	2021 Feb 5	South America / Brazil / Amazonas / Manaus
EPI_ISL_940630	2021 Jan 25	2021 Feb 5	South America / Brazil / Roraima
EPI_ISL_943570	2021 Jan 28	2021 Feb 6	South America / Peru / Lima
EPI_ISL_943967	2021 Jan 25	2021 Feb 7	South America / Brazil / Roraima
EPI_ISL_943968	2021 Jan 25	2021 Feb 7	South America / Brazil / Roraima
EPI_ISL_943969	2021 Jan 25	2021 Feb 7	South America / Brazil / Roraima
EPI_ISL_943970	2021 Jan 25	2021 Feb 7	South America / Brazil / Roraima
EPI_ISL_943971	2021 Jan 25	2021 Feb 7	South America / Brazil / Roraima
EPI_ISL_943987	2021 Jan 20	2021 Feb 7	South America / Brazil / Tocantins / Paraiso do Tocantins
EPI_ISL_943990	2021 Jan 19	2021 Feb 7	South America / Brazil / Amazonas / Manaus
EPI_ISL_981383	2021 Jan 20	2021 Feb 12	South America / Brazil / Sao Paulo / Jau
EPI_ISL_981385	2021 Jan 25	2021 Feb 12	South America / Brazil / Sao Paulo / Jau
EPI_ISL_981387	2021 Jan 18	2021 Feb 12	South America / Brazil / Sao Paulo / Jau
EPI_ISL_983865	2021 Feb 1	2021 Feb 12	South America / Brazil / Rio Grande do Sul / Gramado

Sample identification	Date collected	Date uploaded	Collection locations
EPI_ISL_984619	2021 Feb 3	2021 Feb 13	South America / Brazil / Rio Grande do Sul
EPI_ISL_985303	2021 Jan 19	2021 Feb 15	South America / Brazil / Goias / Aparecida de Goiania
EPI_ISL_985304	2021 Jan 19	2021 Feb 15	South America / Brazil / Goias / Aparecida de Goiania
EPI_ISL_985305	2021 Jan 19	2021 Feb 15	South America / Brazil / Goias / Aparecida de Goiania
EPI_ISL_985306	2021 Jan 19	2021 Feb 15	South America / Brazil / Goias / Aparecida de Goiania
EPI_ISL_985307	2021 Jan 19	2021 Feb 15	South America / Brazil / Goias / Aparecida de Goiania
EPI_ISL_985308	2021 Jan 19	2021 Feb 15	South America / Brazil / Goias / Aparecida de Goiania
EPI_ISL_985309	2021 Jan 19	2021 Feb 15	South America / Brazil / Goias / Aparecida de Goiania
EPI_ISL_985310	2021 Jan 19	2021 Feb 15	South America / Brazil / Goias / Aparecida de Goiania
EPI_ISL_985311	2021 Jan 19	2021 Feb 15	South America / Brazil / Goias / Aparecida de Goiania
EPI_ISL_985312	2021 Jan 19	2021 Feb 15	South America / Brazil / Goias / Aparecida de Goiania
EPI_ISL_985313	2021 Jan 19	2021 Feb 15	South America / Brazil / Goias / Aparecida de Goiania
EPI_ISL_985314	2021 Jan 19	2021 Feb 15	South America / Brazil / Goias / Aparecida de Goiania
EPI_ISL_985315	2021 Jan 19	2021 Feb 15	South America / Brazil / Goias / Aparecida de Goiania
EPI_ISL_985316	2021 Jan 19	2021 Feb 15	South America / Brazil / Goias / Aparecida de Goiania
EPI_ISL_985317	2021 Jan 19	2021 Feb 15	South America / Brazil / Goias / Aparecida de Goiania
EPI_ISL_985318	2021 Jan 8	2021 Feb 15	South America / Brazil / Santa Catarina / Florianopolis
EPI_ISL_985319	2021 Jan 12	2021 Feb 15	South America / Brazil / Santa Catarina / Florianopolis

*SARS-CoV-2, severe acute respiratory syndrome coronavirus 2.

Appendix 1 Table 3. Summary of 6 clades of severe acute respiratory syndrome coronavirus 2 found by using variant of concern PCR genomic surveillance, Uruguay*

Clade	No. isolates	Departments	No. sequences per site	T_{MRCA}, date 2021 (95% CI)	No. syn sites
UY-I	24	Artigas	2	15 Feb (31 Jan–27 Feb)	1†
		Canelones	1		
		Montevideo	5		
		Rio Negro	11		
		Salto	2		
		San Jose	1		
		Soriano	2		
UY-II	12	Canelones	2	17 Feb (23 Jan–4 Mar)	4
		Colonia	1		
		Montevideo	2		
		Rio Negro	5		
		San Jose	2		
UY-III	7	Canelones	4	1 Mar (20 Feb–5 Mar)	6†
		Montevideo	3		
UY-IV	4	Canelones	1	28 Feb (19 Feb–5 Mar)	1
		Soriano	1		
		San Jose	2		
UY-V	3	Rocha	3	21 Feb (Feb 12–23)	2†
UY-VI	3	Rocha	3	15 Feb (Feb 1–22)	2†

*syn, synapomorphic sites defining each clade; T_{MRCA}, time of most common recent ancestor.
†Amino acid replacement is considered radical. See Appendix Table 4 for additional details on the clades and synapomorphies.

Appendix 1 Table 4. Introductions of SARS-CoV-2 P.1 lineage and synapomorphic sites defining each clade, Uruguay*

Clade	No. isolates	Department (no. samples)	T_{MRCA}, date 2021 (95% CI)†	Genomic region	Nucleotide	Amino acid	Amino acid replacement
UY-I	24	AR (2), CA (1), MO (5), RN (11), SA (2), SJ (1), SO (2)	15 Feb (31 Jan–27 Feb)	ORF1a (NSP3)	G8102T	V1795F	radical
UY-II	12	CA (2), CO (1), MO (2), RN (5), SJ (2)	17 Feb (23 Jan–4 Mar)	ORF1a (NSP3)	A3957G	K413R	conservative
				M	T26597C	G25G	–
				ORF8	T27986C	Y31Y	–
				3'UTR	G29760A	–	–
UY-III	7	CA (4), MO (3)	01 Mar (20 Feb–5 Mar)	ORF1a (NSP3)	C5310T	T864I	radical
				ORF1a (NSP4)	G8849T	V99L	conservative
				ORF1a (NSP5)	C10507T	N151N	–
				ORF1a (NSP6)	C11074T	F34F	–
				ORF3a	T26171A	M260K	radical
				Intergenic	C27389A	–	–
UY-IV	4	CA, SJ (2), SO (1)	28 Feb (19 Feb–5 Mar)	ORF3a	A25450G	I20V	conservative
UY-V‡	3	RO (3)	21 Feb (12–23 Feb)	ORF1b (NSP13)	G17797A	V521I	conservative
				Spike	G22471T	L303F	radical
UY-VI	3	RO (3)	15 Feb (1–22 Feb)	ORF1a (NSP5)	A10323G	K90R	conservative
				ORF1a (NSP6)	C11001T	T10I	radical
UY-VII	1	AR (1)	–	–	–	–	–
UY-VIII	1	AR (1)	–	–	–	–	–
UY-VIIII	1	MO (1)	–	–	–	–	–
UY-X	1	RN (1)	–	–	–	–	–
UY-XI	1	SA (1)	–	–	–	–	–
UY-XII	1	TA (1)	–	–	–	–	–

*We provide a description of the synapomorphies defining each clade that had ≥3 samples. AR, Artigas; CA, Canelones; COVID-19, Coonia; MO, Montevideo; NSP, nonstructural protein; ORF, open reading frame; RN, Rio Negro; RO, Rocha; SA, Salto; SJ, San José; SO, Soriano; TA, Tacuarembó; T_{MRCA}, time of most recent common ancestor; SARS-CoV-2, severe acute respiratory syndrome coronavirus 2; –, not applicable.
†T_{MRCA} was estimated by using BEAST (*27*).
‡Clade UY-V is defined by two synapomorphies, 1 being the nonsynonymous S:L303F change in the spike protein. This change has been reported before, in 41 sequences from Europe and the United States, mainly in B.1.1.7 sequences, and involves a radical replacement regarding amino acid volume and aromaticity. Other radical changes we described have not been reported in South America before, except for NSP3:T864I, a radical replacement in polarity, previously found in Brazil and Chile; and NSP3:V1795F a radical replacement in volume and aromaticity previously found in Brazil.

Appendix 1 Table 5. Primers and probes designed for ORF1ab drop-out PCR for SARS-CoV-2 variants of concern, Uruguay*

Name	Sequence
ORF1a_11247–11267_Fw	TGCGTATTATGACATGGTTGG
ORF1a_11369–11388_Rv	ACTCTCCTAGCACCATCATC
ORF1a_11272–11300_Pb	/5Cy5/GGTTGATAC/TAO/TAGTTTGTCTGGTTTTAAGC/3IAbRQSp/
S_Fw	TCAACTCAGGACTTGTTCTTACCT
S_Rv	TGGTAGGACAGGGTTATCAAAC
S_Pb	/5HEX/TTCCATGCT/ZEN/ATACATGTCTCTGGGA/3IABkFQ/

*ORF, open reading frame; SARS-CoV-2, severe acute respiratory syndrome coronavirus 2.

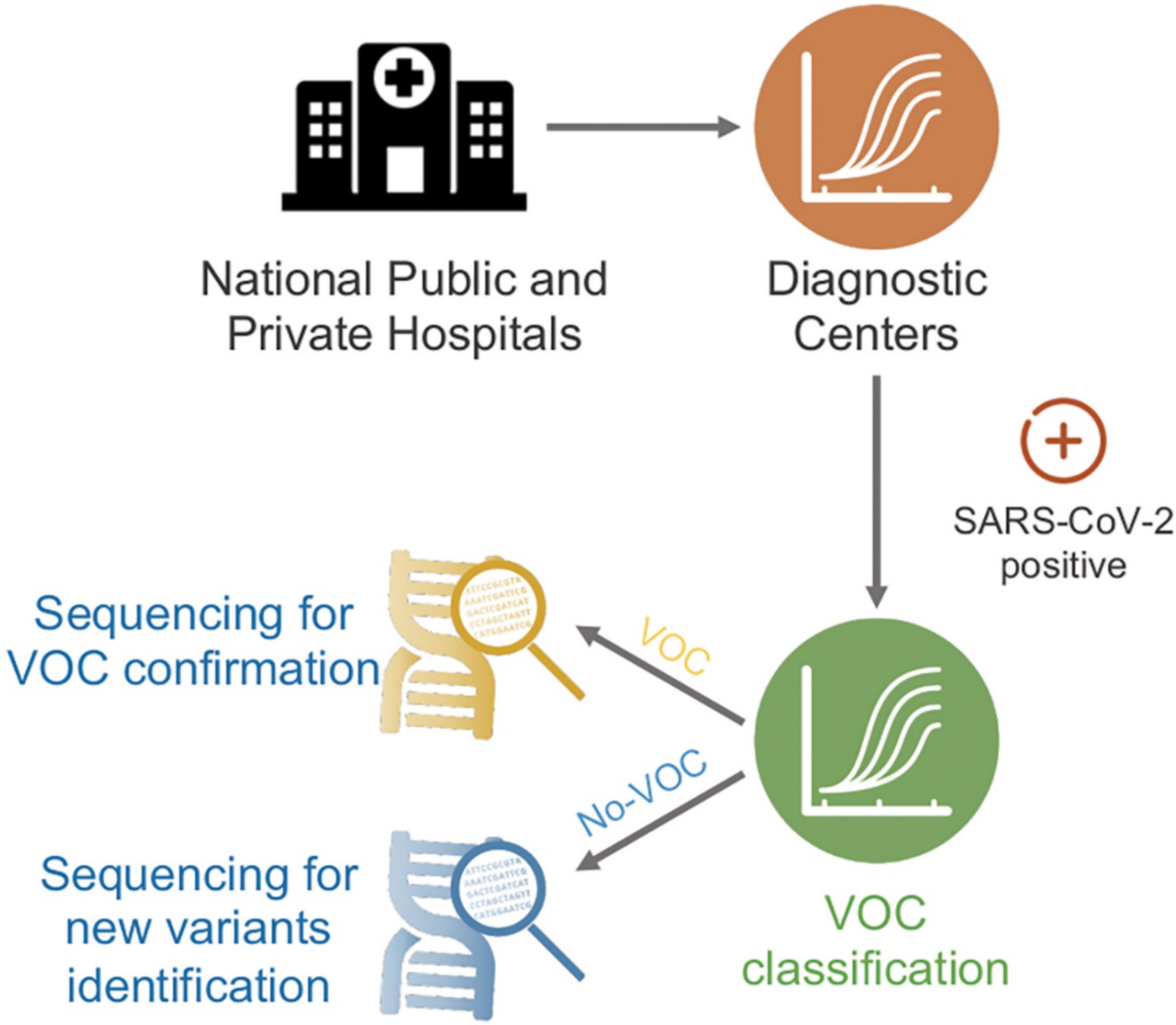

Appendix 1 Figure 1. Interinstitutional working group workflow for identifying SARS-CoV-2 variants of concern (VOCs), Uruguay. Diagnostic centers performed standard PCR for identification of SARS-CoV-2–positive samples, which are further evaluated by a drop-out PCR assay to determine known VOCs. A subset of samples classified as VOC and no-VOC by drop-out PCR then are sequenced to confirm known VOCs and detect new potential variants. SARS-CoV-2, severe acute respiratory syndrome coronavirus 2.

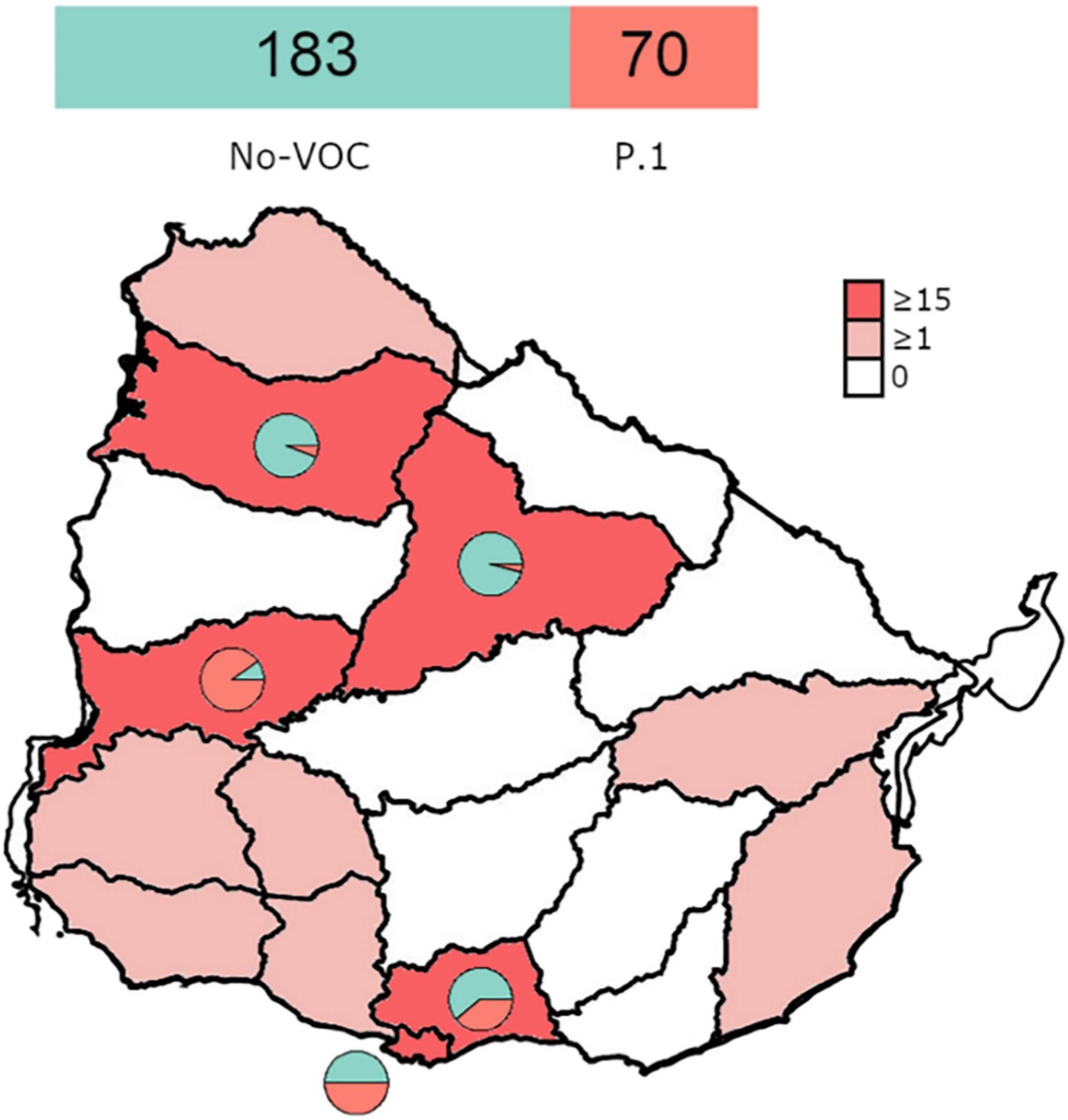

Appendix 1 Figure 2. Barplot showing proportions of SARS-CoV-2 variant P.1 and no variants of concern (no-VOCs), Uruguay. Total numbers of samples in each category are shown. Shading in map shows location in which P.1 was detected; dark red indicates ≥15 P.1–positive samples; light red shading indicates 1–14 P.1–positive samples. Pie charts indicate proportions of VOC and no-VOCs in departments with ≥15 samples. SARS-CoV-2, severe acute respiratory syndrome coronavirus 2.

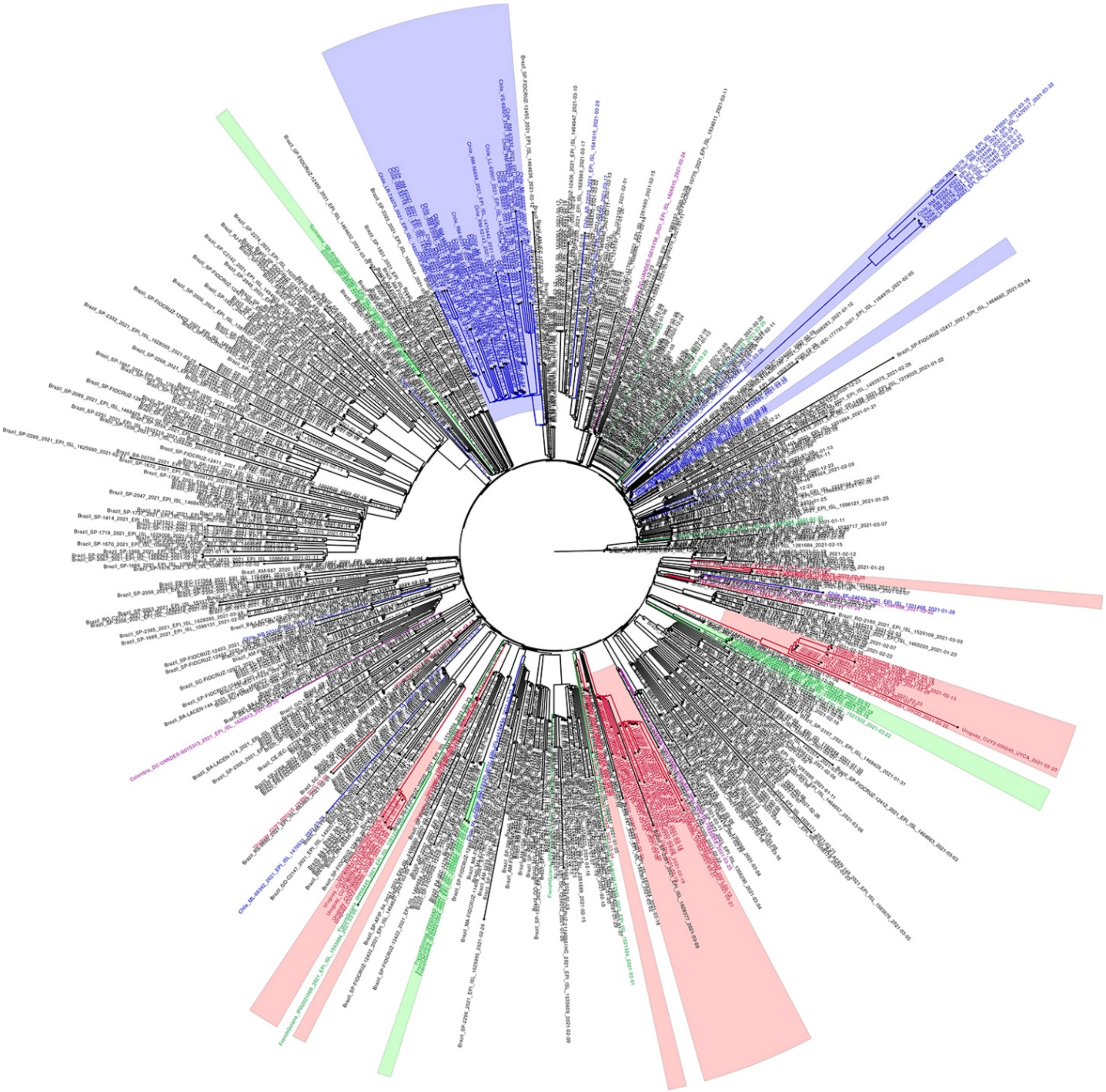

Appendix 1 Figure 3. Maximum-likelihood phylogenetic tree of 59 SARS-CoV-2 P.1 whole genome sequences from Uruguay and 691 from South America. The tree was rooted with the oldest P.1 sequence (GISAID accession no. EPI_ISL_833137). Red text indicates sequences from Uruguay. Black text indicates sequences from Brazil. Blue text indicates sequences and clades from Chile. Green text indicates sequences and clades from French Guiana and Suriname. Violet text indicates sequences from Colombia and Peru. Among P.1 sequences, 6 were singletons, but 53 sequences gather in 6 clades of 3–24 sequences (red shading). SARS-CoV-2, severe acute respiratory syndrome coronavirus 2.

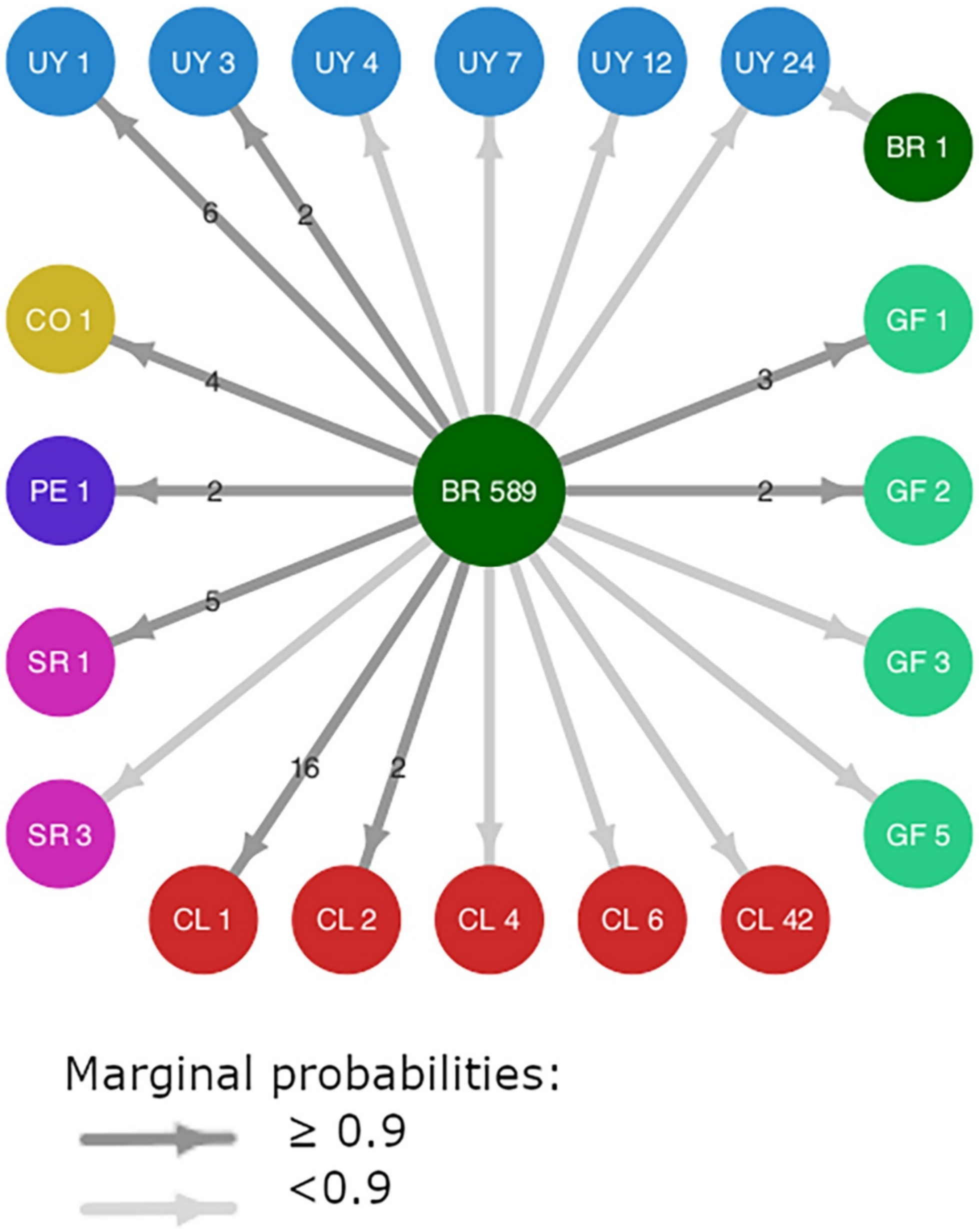

Appendix 1 Figure 4. Schematic representation of migration events for SARS-CoV-2 P.1 variant of concern (VOC), Uruguay. Each node in the network is identified by location and number of sequences within different phylogenetic subclusters. Arrows indicate migration events deduced from location state changes across the tree. Marginal probabilities are represented by different shades of gray arrows and the numbers quantify the migration events connecting respective locations; no number represents a single event. Nodes are colored according to their location. BR, Brazil; CL, Chile; COVID-19, Colombia; GF, French Guayana; PE, Peru; SARS-CoV-2, severe acute respiratory syndrome coronavirus 2; SR, Surinam; UY, Uruguay.

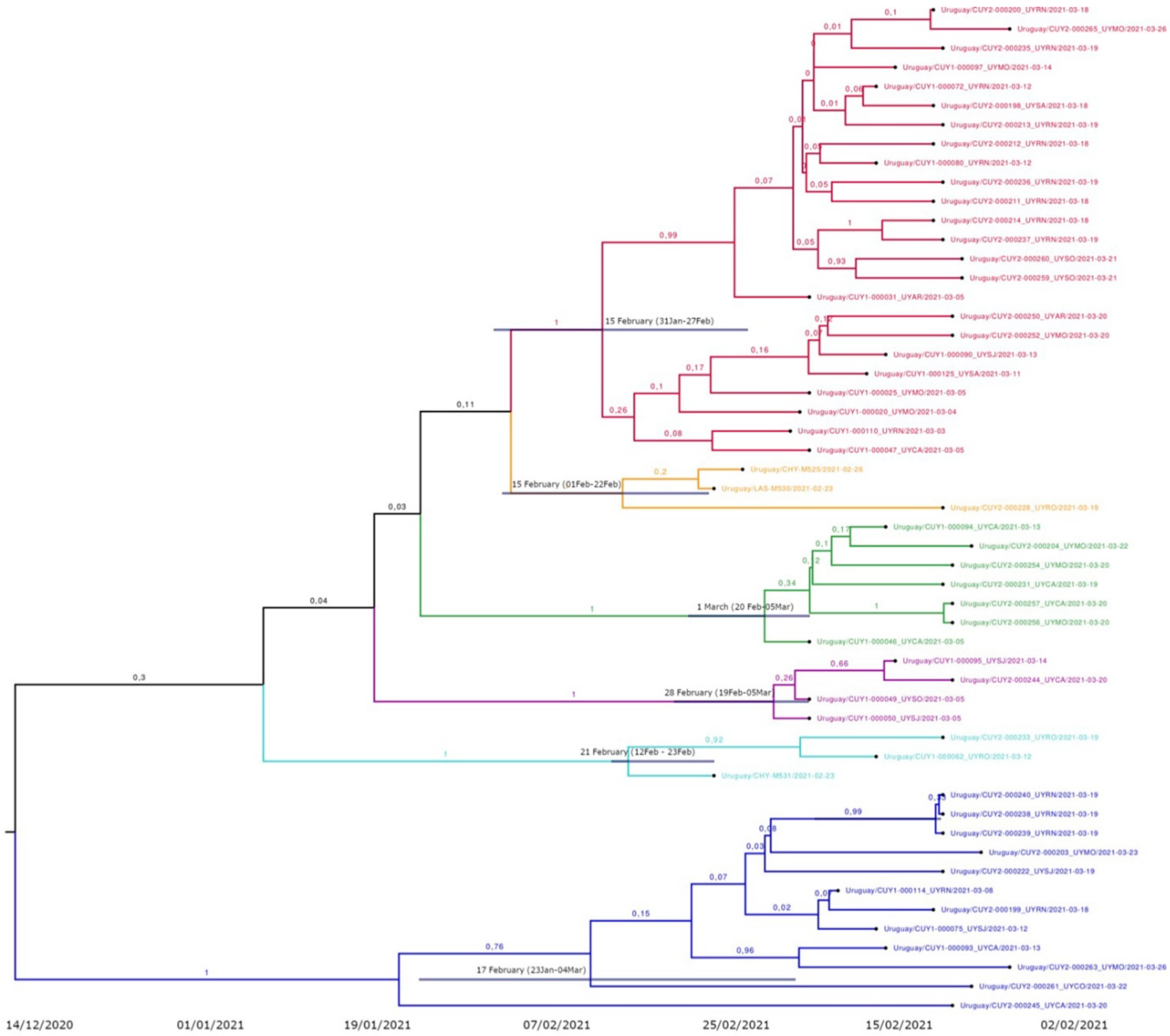

Appendix 1 Figure 5. Temporal dissemination of SARS-CoV-2 clades I–VI, Uruguay (UY). Six clades from Uruguay are inferred on the time-scaled Bayesian phylogeographic maximum clade credibility tree. Time of most common recent ancestor and 95% CIs are shown at ancestral nodes. Red indicates UY-I; blue indicates UY-II; green indicates UY-III; violet indicates UY-IV; aquamarine indicates UY-V; and yellow indicates UY-VI.

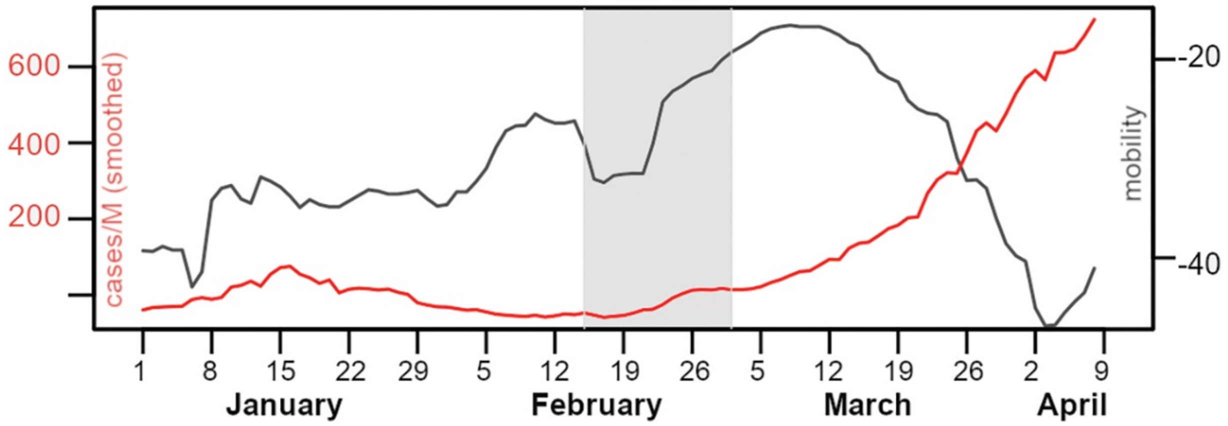

Appendix 1 Figure 6. Mobility index and number of SARS-CoV-2 cases per million persons, Uruguay. Black indicates mobility index; red indicates number of cases per million persons daily. We used methods from Report 11 of the Uruguayan Interdisciplinary Group for COVID-19 Data Analysis (GUIAD-COVID-19)-Feb 2021 (https://hdl.handle.net/20.500.12008/27166) to calculate mobility index. The time ranges from January 1–April 9, 2021. Gray shading indicates the estimated period in which P.1 most likely was introduced to the country (≈February 15, 2021).
